# Quasi-Static Penetration Resistance of Bio-Inspired Helicoidal Honeycomb Sandwich Panels: Experiments, Simulations, and Damage Mechanisms

**DOI:** 10.3390/ma19173778

**Published:** 2026-09-04

**Authors:** Xin Du, Xin Lian, Chunhua Wan, Zhefeng Yu

**Affiliations:** 1 Aerospace Structure Research Center, School of Aeronautics and Astronautics, Shanghai Jiao Tong University, Shanghai 200240, China; xindu_sjtu@sjtu.edu.cn; 2 Aerospace System Engineering Shanghai, Shanghai 200240, China; 412146266@alumni.sjtu.edu.cn; 3 National Key Laboratory of Strength and Structural Integrity, Aviation Key Laboratory of Science and Technology on Structures Impact Dynamics, Aircraft Strength Research Institute of China, Xi’an 710065, China; wanc_1@163.com; 4 State Key Laboratory of Metal Matrix Composites, Shanghai 200240, China

**Keywords:** Bouligand structures, helicoidal laminate, Nomex honeycomb sandwich panel, quasi-static penetration, progressive damage

## Abstract

Bio-inspired helicoidal laminates can redirect damage under transverse loading, but their response as sandwich face sheets remains unclear because the core changes both deformation and load transfer. This study experimentally and numerically investigated the quasi-static penetration of 73-ply carbon/epoxy laminates and Nomex honeycomb sandwich panels with cross-ply, quasi-isotropic, uniform helicoidal (5°, 10°, and 20°), and hybrid helicoidal layups. A fully ply-resolved model was developed for the monolithic laminates, whereas an eight-sublaminate model with an explicitly represented honeycomb core was used for the sandwich panels. H73(10–5) achieved the highest monolithic-laminate peak load of 5.23 kN, 46.1% above CP73 and 35.5% above QI73. The sandwich panels exhibited two load peaks separated by a core-crushing plateau. S-H73(5–10) produced the highest first peak load of 8.99 kN, while S-H73(10–5) achieved the highest penetration energy of 108.37 J. Experiments, simulations, and fractographic observations showed that the intact core constrained upper-face-sheet bending and promoted localized indentation-assisted punching-shear perforation. The crushed core subsequently transferred load to the lower face sheet, which failed through bending- and membrane-dominated tearing.

## 1. Introduction

Lightweight sandwich structures offer high specific stiffness, high specific strength, thermal and acoustic insulation, and considerable design flexibility [1]. They are widely used in aerospace, transportation, marine, construction, energy, and protective engineering applications. A conventional sandwich panel consists of two thin and stiff face sheets bonded to a lightweight core. The face sheets primarily carry bending-induced normal stresses, whereas the core maintains the separation between the face sheets and resists transverse shear and through-thickness compression [2,3]. This structural arrangement provides a much higher bending stiffness-to-weight ratio than a monolithic plate of comparable mass.

Common core materials include Nomex paper honeycombs [4], aluminum cellular and lattice structures [5,6], and polymeric or metallic foams [7]. Recent developments also include flexible core materials that serve as infusion media and additively manufactured sandwich-core inserts or related core-integrated components [8,9]. Despite their high structural efficiency, sandwich panels are vulnerable to concentrated transverse loading because their face sheets are relatively thin [1,10]. Local indentation can initiate matrix cracking, fiber fracture, delamination, core crushing, and eventual face-sheet penetration. Once penetration occurs, the residual stiffness and load-carrying capacity decrease, while the exposed cellular core may become susceptible to moisture ingress and subsequent degradation [11]. The local penetration resistance of the face sheets is therefore an important consideration in sandwich-panel design.

Paper-based cellular panels provide an industrially relevant reference for lightweight cushioning and energy absorption. Corrugated paperboard and paper-honeycomb panels are widely used in packaging, furniture, building components, and protective products because of their low density, low cost, and ability to absorb energy through cell-wall bending, buckling, folding, and crushing. Bivainis and Jankauskas [12] showed that the puncture load and puncture energy of corrugated paperboard depend on the paper grade, grammage, flute arrangement, and number of corrugated layers. Wang [13] found that the cushioning and energy absorption responses of paper-honeycomb sandwich panels are strongly influenced by cell dimensions, relative density, core thickness, and liner properties. Wang and Bai [14] further examined the deformation and energy absorption of paper honeycombs under dynamic compression.

Conventional paper-based panels are generally improved by changing the paper grade, core geometry, relative density, liner thickness, or number of layers. Their face sheets are not normally designed to redirect cracks progressively through the thickness after puncture has initiated. Introducing a continuously rotating reinforcement architecture into the face sheets offers a different means of increasing local penetration resistance without increasing face-sheet thickness or mass. Previous studies on engineering sandwich structures have examined changes in both the core and the face sheets. Kepler [10] quantified the effects of panel configuration, impact velocity, and indenter geometry on the penetration and energy absorption of sandwich panels. Qiang et al. [15] showed that fiber-metal-laminate face sheets altered the projectile resistance and tearing behavior of corrugated sandwich structures. Kumar et al. [16] examined the effects of face-sheet thickness and specimen geometry on flexural strength. Qin et al. [17] demonstrated that the thickness distribution between the front and rear face sheets affected the impact damage and energy absorption of aluminum-honeycomb sandwich panels. Zhang et al. [18] further showed that reinforcement orientation in composite fold core panels influenced anisotropic deformation, front-face fracture, rear-face tensile damage, and overall impact resistance. These studies indicate that the reinforcement arrangement of the face sheets should be considered together with the core structure.

A relevant biological design is the Bouligand architecture found in crustacean exoskeletons and other natural protective structures [19,20]. It consists of successive fibrous layers whose orientations rotate gradually through the thickness. Similar helicoidal arrangements have also been identified in beetle cuticles and natural dermal armor [21,22]. When transferred to fiber-reinforced composites, the progressive rotation changes the propagation paths of matrix cracks and delamination fronts. Crack twisting and deflection increase the tortuosity of the fracture path and distribute damage over several fiber orientations [23,24]. Cheng et al. [25] showed that this bio-inspired arrangement can produce mechanical responses distinct from those of conventional laminates.

The mechanical behavior of helicoidal laminates has been investigated under low-velocity impact [19,26], quasi-static transverse loading [19,27,28], three-point bending [24,29], and compression after impact [23,30]. Under low-velocity impact, Ginzburg et al. [19] reported improved damage tolerance and residual performance relative to conventional orthotropic and quasi-isotropic laminates. Mencattelli and Pinho [26] showed that small-angle thin-ply Bouligand structures could promote diffuse subcritical damage and reduce the concentration of severe impact damage. Three-point bending studies examined the roles of crack twisting and progressive damage in the load-bearing response of helicoidal laminates [24,29]. Grunenfelder et al. [23] and Yu et al. [30] also demonstrated that an appropriate helicoidal or hybrid layup can improve residual compressive performance after impact.

Quasi-static transverse loading is particularly relevant to the present study. Shang et al. [31] found that helicoidal laminates with small inter-ply angles could sustain higher transverse loads than cross-ply laminates. Their 19-ply helicoidal configuration exhibited an approximately 34% higher peak load and a predominantly monotonic load–displacement response followed by abrupt failure. Delamination propagated between successive interfaces and developed into a spiral pattern, in contrast to the multiple delaminations observed in the cross-ply specimens. Liu et al. [27] subsequently showed that major load drops were governed by the interaction between transverse cracks growing from the tensile surface and delamination within the laminate. The cross-ply specimens contained several major delaminations and exhibited multiple load drops, whereas the helicoidal laminates reached a higher peak load before a single catastrophic reduction in load. The response of a helicoidal laminate does not improve monotonically as the inter-ply angle decreases. Liu et al. [28] showed that the rotation angle controls the competition among matrix splitting, delamination, and fiber damage. Smaller angles delay the formation of multiple delamination but allow matrix splits to twist over a greater distance. Larger angles can restrict matrix splitting but tend to increase interlaminar mismatch and delamination. Mencattelli and Pinho [26,29] similarly demonstrated that the beneficial response of thin-ply Bouligand laminates depends on the development of distributed subcritical damage rather than on minimizing the pitch angle without limit.

Natural Bouligand structures may also exhibit through-thickness variations in pitch. Grunenfelder et al. [23] reported a gradual variation in the helicoidal arrangement of the mantis-shrimp impact region. Motivated by this feature, Liu et al. [32] tested 73-ply carbon/epoxy laminates with non-uniform inter-ply angles. Matrix splitting initiated mainly near the lower tensile surface, where a relatively larger inter-ply angle improved resistance to splitting. Delamination was concentrated closer to the loading region and was mitigated by a smaller inter-ply angle.

Related studies have extended helicoidal concepts and composite-impact methods to other structural configurations. Lian et al. [33] developed a curved grid-stiffened helicoidal composite panel and reported weight reductions of 13.26% and 11.68% relative to an angle-grid design under uniaxial compression and shear loading, respectively. Yu and co-workers investigated strain-rate-dependent low-velocity impact response [34], contact-radius effects and delamination-threshold prediction [35], and the impact damage resistance of a co-cured composite wing box [36]. These studies do not all concern helicoidal laminates directly, but they provide relevant analytical and numerical methods for composite impact assessment. Sasikumar et al. [37] also showed that an unsymmetrical thin-ply design containing intermediate-grade plies could improve the impact response of thin laminates.

Most previous Bouligand studies have focused on monolithic laminates. Much less information is available on helicoidal laminates used as face sheets in cellular sandwich panels. Their response cannot be inferred directly from monolithic-plate tests because the core provides distributed support to the impacted face sheet and changes its flexural deformation, local indentation, and subsequent load transfer to the rear face sheet. The fold core results reported by Zhang et al. [18] confirm that the interaction between structural support and reinforcement orientation can modify the damage modes of both the front and rear surfaces. However, constant-pitch and through-thickness-varying helicoidal face sheets have not been systematically compared in Nomex honeycomb sandwich panels under quasi-static penetration.

Experiments provide the global load–displacement response and the final damage morphology, but they cannot fully resolve the development of matrix damage, fiber failure, interlaminar delamination, and honeycomb-cell crushing inside the panel. Classical impact models have long demonstrated the importance of representing intralaminar damage [38], while more recent studies have considered the influence of delamination on the global response of composite plates [39]. Finite element analysis is therefore useful for interpreting the internal damage sequence, but its predictions depend on the failure criterion, damage-evolution law, and interface representation. Huang et al. [40] assessed Hashin and Puck initiation criteria, three damage-evolution methods, and three cohesive-interface treatments for thin composite laminates under low-velocity impact. Their results showed that the damage-evolution method had a stronger influence on the calculated response than the difference between the two initiation criteria. The interface representation also affected the predicted damage. These findings indicate that a numerical model should be validated against both the measured mechanical response and the observed damage, rather than only against a single peak-load value.

The present study investigates the quasi-static penetration behavior of 73-ply carbon/epoxy helicoidal laminates and Nomex honeycomb sandwich panels through experiments and finite element simulations. Monolithic cross-ply, quasi-isotropic, and helicoidal laminates are first tested under the same concentrated-loading condition. Sandwich panels manufactured with the corresponding face sheets are then examined to determine how the honeycomb core changes the stiffness, peak loads, failure displacement, penetration energy, and damage sequence. Constant inter-ply angles of 5°, 10°, and 20° are considered together with the hybrid H73(10–5), H73(5–10), H73(10–20), and H73(20–10) configurations.

A two-level finite element strategy is adopted. A ply-resolved model consisting of SC8R continuum-shell plies and COH3D8 cohesive interfaces is used to examine fiber and matrix damage and ply-level delamination in the monolithic laminates. A sub-laminate representation is used for the complete sandwich panels to reduce computational cost while retaining the through-thickness variation in fiber orientation. The simulations are validated using the measured load–displacement curves and observed damage morphologies and are subsequently used to interpret face-sheet failure, honeycomb crushing, and load transfer during penetration. The study therefore extends helicoidal design from monolithic laminates to honeycomb sandwich face sheets, compares constant and hybrid inter-ply-angle distributions, and combines experiments and numerical analysis to clarify the associated penetration and energy-absorption mechanisms.

The remainder of this paper is organized as follows. Section 2 describes the specimen configurations, manufacturing procedure, quasi-static penetration tests, and finite element modeling method. Section 3 presents the experimental and numerical results for the monolithic laminates and sandwich panels and discusses their damage and energy-absorption mechanisms. Section 4 summarizes the main conclusions.

## 2. Experimental and Numerical Methods

### 2.1. Specimen Configurations

**Figure 1:**
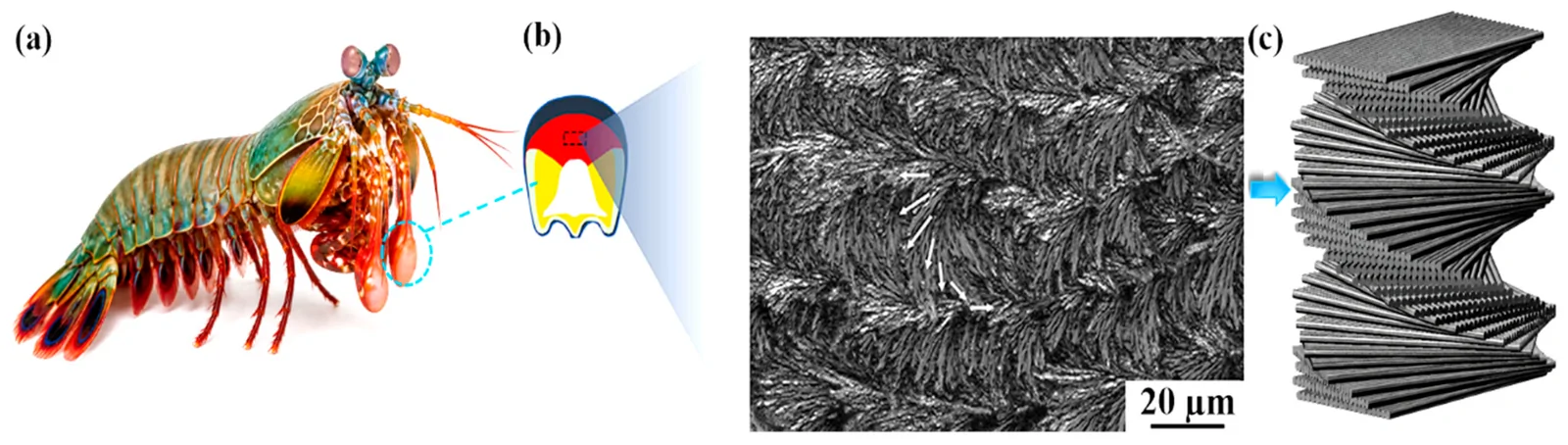
Biological inspiration and structural abstraction of the Bouligand laminate: (**a**) a mantis shrimp and its hammer-like dactyl club; (**b**) morphology of *Odontodactylus japonicus*: SEM image of the cross-section of the periodic region (marked in red). The SEM micrograph was adapted from Yin et al. [41] under the Creative Commons Attribution 4.0 license; and (**c**) the corresponding small-pitch-angle carbon-fiber-reinforced polymer laminate constructed by progressively rotating the fiber orientation of adjacent unidirectional plies.

The periodic region of the mantis shrimp dactyl club consists of mineralized chitin–protein lamellae arranged in a Bouligand architecture, where the in-plane fibril direction progressively rotates by a small angle between adjacent layers [41]. This natural helicoidal organization has inspired the development of damage-tolerant composite laminates. In this study, the biological lamellae are abstracted into unidirectional carbon/epoxy plies, and a small rotation angle is introduced between adjacent plies to construct the helicoidal CFRP face-sheet configurations investigated herein, as illustrated in Figure 1.

**Figure 2:**
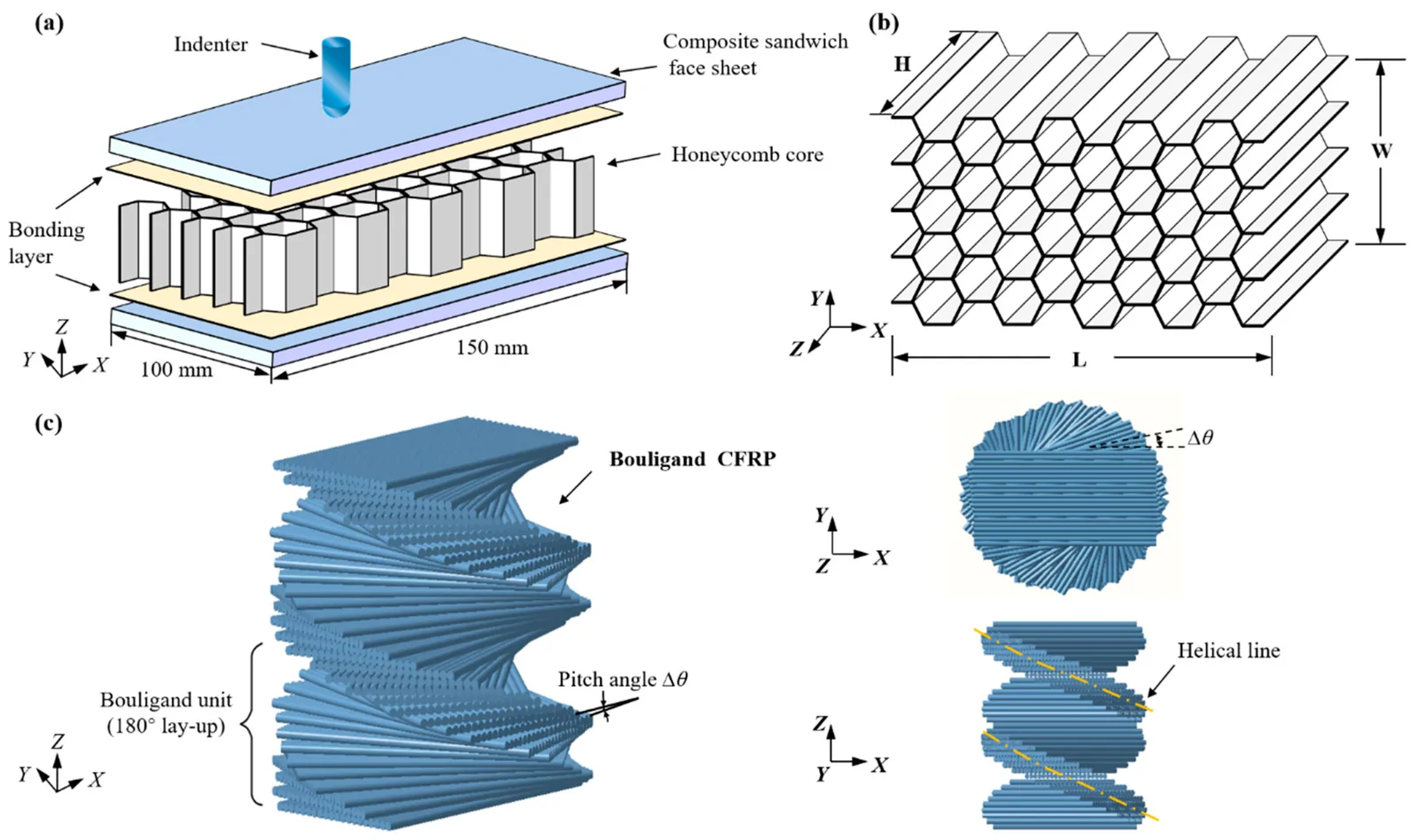
Schematic of the Bouligand-structured composite sandwich panel: (**a**) geometry of the Nomex honeycomb sandwich structure under out-of-plane loading; (**b**) hexagonal meta-aramid paper honeycomb core, where H, W, and L denote the height, width, and length of the honeycomb core, respectively; and (**c**) Bouligand-inspired helicoidal layup of the composite face sheet.

Based on this design concept, bio-inspired honeycomb sandwich panels were constructed using helicoidal carbon-fiber-reinforced composite laminates as the upper and lower face sheets and a hexagonal meta-aramid paper honeycomb as the core, as schematically shown in Figure 2. In this study, the notation H73 denotes a 73-ply helicoidal laminate used as a face sheet, whereas S-H73 denotes the corresponding sandwich panel incorporating two H73 face sheets and a honeycomb core. The helicoidal face sheets were manufactured from unidirectional T700/SKRK51 carbon/epoxy prepreg. Different through-thickness inter-ply angle distributions were designed to examine the effects of both uniform and non-uniform helicoidal layups on the quasi-static penetration response of the sandwich panels. Table 1 summarizes the laminate and sandwich-panel configurations investigated in this study, together with the abbreviations used throughout the paper.

**Figure 3:**
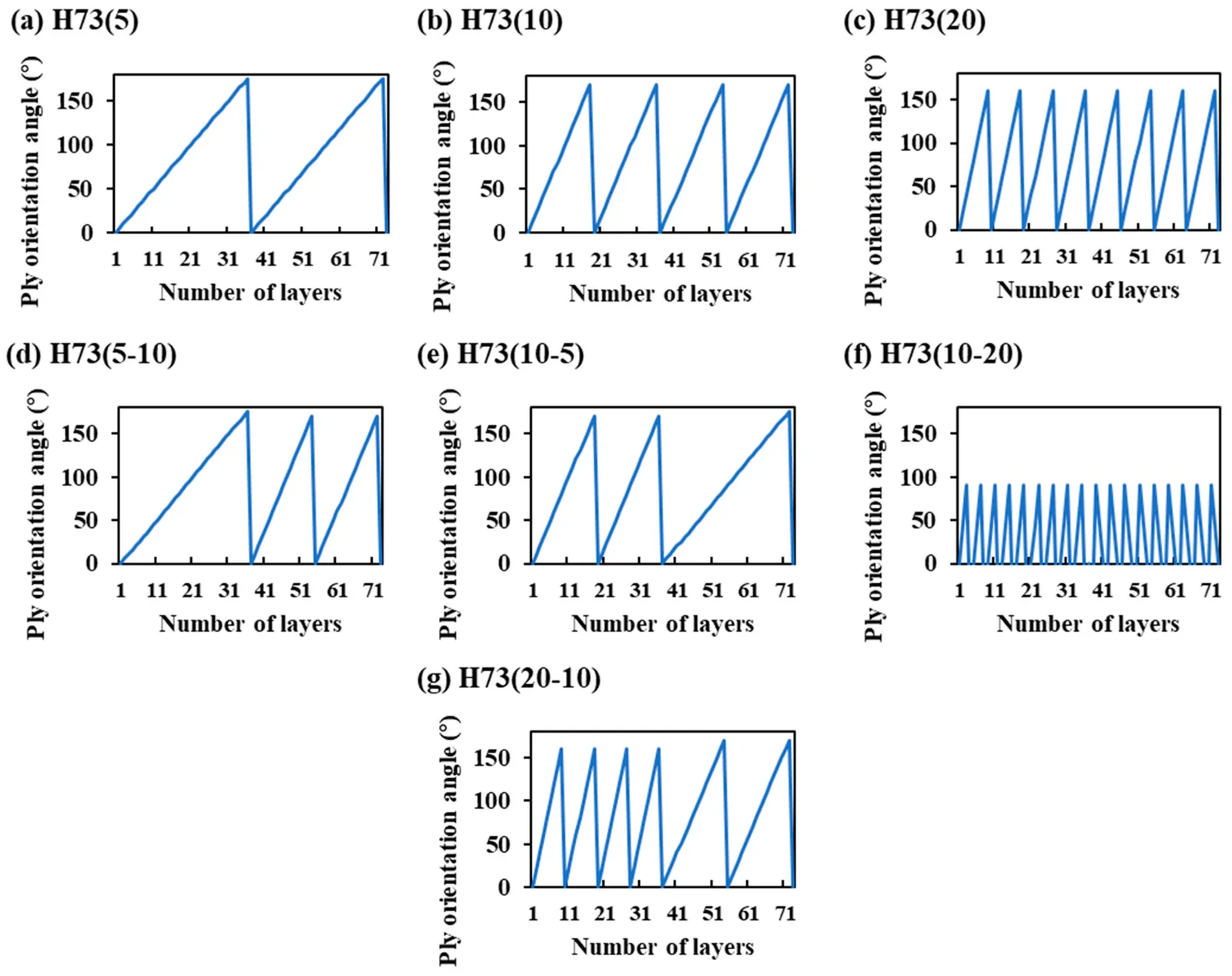
Through-thickness ply-orientation profiles of the helicoidal face-sheet laminates: (**a**) H73(5); (**b**) H73(10); (**c**) H73(20); (**d**) H73(5–10); (**e**) H73(10–5); (**f**) H73(10–20); and (**g**) H73(20–10).

For the uniform helicoidal configurations, the inter-ply angle was kept constant through the thickness of the face sheet. For example, H73(5), H73(10), and H73(20) denote 73-ply helicoidal laminates with constant inter-ply angles of 5°, 10°, and 20°, respectively. For the non-uniform configurations, the inter-ply angle was varied stepwise between the lower and upper halves of the face sheet. Specifically, H73(10–5) denotes a laminate with a 10° inter-ply angle in the lower half and a 5° inter-ply angle in the upper half along the thickness direction. This design was motivated by the graded Bouligand architecture observed in crustacean exoskeletons [23], where the local rotation rate of fibrous layers varies through the thickness. H73(5–10) was obtained by reversing the through-thickness order of H73(10–5), allowing the influence of the inter-ply angle distribution relative to the loading surface to be evaluated. Additional hybrid configurations, such as H73(10–20) and H73(20–10), were also included to further examine the effect of placing larger inter-ply angles in different thickness regions. Quasi-isotropic (QI) and cross-ply (CP) face-sheet laminates were fabricated as baseline configurations for comparison. The ply-orientation profiles of the different face-sheet laminates are illustrated in Figure 3. These profiles clarify the distinction between uniform helicoidal layups, in which the inter-ply angle remains constant, and hybrid helicoidal layups, in which the inter-ply angle changes through the thickness. Unless otherwise specified, the ply numbering direction was defined consistently from the lower surface to the upper surface of each face sheet.

Individual prepreg plies were manually laid up according to the prescribed stacking sequences and cut into rectangular specimens with in-plane dimensions of 150 mm × 100 mm. These dimensions were selected following the specimen size commonly used for out-of-plane impact and penetration testing of composite laminates [42,43]. During lay-up, the prescribed ply orientations were controlled using an angle-indexed positioning template, whose orientation was set and checked using a digital angle rule before placement of each prepreg ply. The unidirectional prepreg was aligned with the corresponding reference direction on the template to maintain the prescribed stacking sequence and inter-ply angles. The 73-ply face sheets were cured in an autoclave at 125 °C under a pressure of 5 bar for 90 min, followed by cooling to room temperature over 10 h. The cured face sheets had an average ply thickness of approximately 30 μm and an overall thickness of approximately 2.2 mm. The sandwich core was an AC-NH meta-aramid honeycomb (AC-NH-1.83–128) with a nominal cell side length of 1.83 mm, a density of 128 kg/m^3^, a core height of 20 mm, and a nominal out-of-plane compressive strength of 10 MPa. The upper and lower carbon/epoxy helicoidal composite face sheets were bonded to the aramid honeycomb core using an epoxy-based structural adhesive during fabrication of the sandwich panels. The total thickness of the sandwich panels was approximately 24.4 mm. Detailed thickness information for the face sheets, honeycomb core, and assembled sandwich panels is provided in Table 2.

### 2.2. Quasi-Static Penetration Test Setup and Procedure

Quasi-static out-of-plane penetration tests were conducted to characterize the load-bearing capacity and damage resistance of the monolithic laminates and honeycomb sandwich panels. The experimental procedure was based on the edge-supported quasi-static indentation provisions of ASTM D6264/D6264M [43] and ASTM D7766/D7766M [44], together with concentrated transverse-loading configurations used in previous studies on helicoidal laminates and sandwich structures [21,35]. To ensure direct comparability, the monolithic laminates listed in Table 1 were tested using the same loading and support configuration as the sandwich panels.

**Figure 4:**
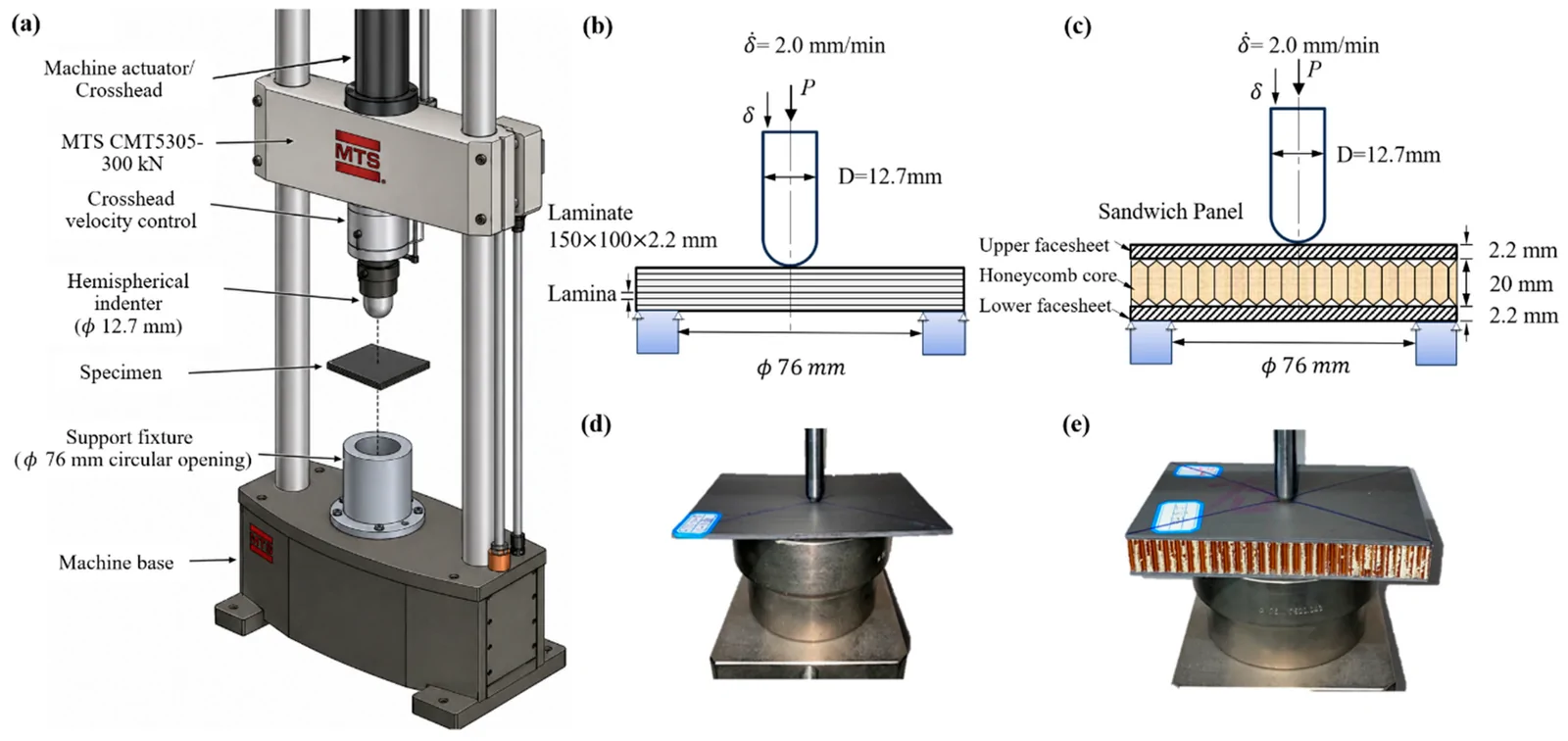
Experimental setup and schematic illustration of the quasi-static penetration tests: (**a**) isometric view of the testing machine, hemispherical indenter, specimen, and support fixture; (**b**) cross-sectional schematic of the monolithic-laminate test; (**c**) cross-sectional schematic of the honeycomb sandwich-panel test, showing the loading direction, 12.7 mm-diameter hemispherical indenter, 76 mm-diameter support opening, and specimen dimensions; (**d**) photograph of the monolithic-laminate test setup; and (**e**) photograph of the honeycomb sandwich-panel test setup.

As illustrated in Figure 4, the specimens were centrally positioned on a rigid hollow steel cylinder with a circular opening of 76 mm in diameter. The support fixture provided an out-of-plane constraint that was independent of the in-plane fiber orientation, while allowing the specimens to deform through a coupled response of local indentation and global bending under transverse loading. This fixture configuration is therefore suitable for assessing the penetration resistance of anisotropic composite laminates and sandwich panels. In particular, the unsupported span defined by the 76 mm opening allowed the lower surface of the specimen to deform freely, which is important for the development of representative failure modes such as bottom-face cracking, interlaminar delamination, and, in the case of sandwich panels, honeycomb core crushing. The load was applied at the geometric center of each specimen through a hemispherical steel indenter with a diameter of 12.7 mm. The indenter was mounted on a universal testing machine (CMT5305-300 kN, MTS Systems, Shanghai, China), and all tests were performed under displacement control at a constant crosshead speed of 2.0 mm/min to ensure a stable quasi-static response. Load and displacement were recorded continuously throughout the test to obtain the load–displacement response. The key performance metrics extracted from the load–displacement curves included the initial stiffness, peak load, failure displacement, and penetration energy. The penetration energy $E_{p}$ was calculated as the area under the load–displacement curve up to the failure point. Consistent with previous studies [30], failure was defined as a 30% reduction from the peak load. This criterion was applied consistently to both the monolithic laminates and the honeycomb sandwich panels to enable a comparative evaluation of their quasi-static penetration resistance.

In addition to the penetration energy, the specific energy absorption (SEA) and energy absorption efficiency were introduced to evaluate the mass-normalized and load- utilization characteristics of the specimens. SEA was calculated as

(1) $$SEA=\frac{E_{p}}{m}$$

where $E_{p}$ is the penetration energy and m is the measured specimen mass. Since the penetration damage is highly localized beneath the indenter, the calculated SEA represents a specimen-level comparative indicator under the present test configuration rather than an intrinsic energy-absorption property of the material system. The energy absorption efficiency was defined as

(2) $$\eta_{E}=\frac{E_{p}}{F_{max}\delta_{f}}\times 100\%$$

where $F_{max}$ is the maximum load and $\delta_{f}$ is the displacement corresponding to the endpoint used for calculating $E_{p}$. This quantity represents the ratio of the actual absorbed energy to the ideal rectangular energy over the same displacement range.

### 2.3. Finite Element Modeling Strategy

**Figure 5:**
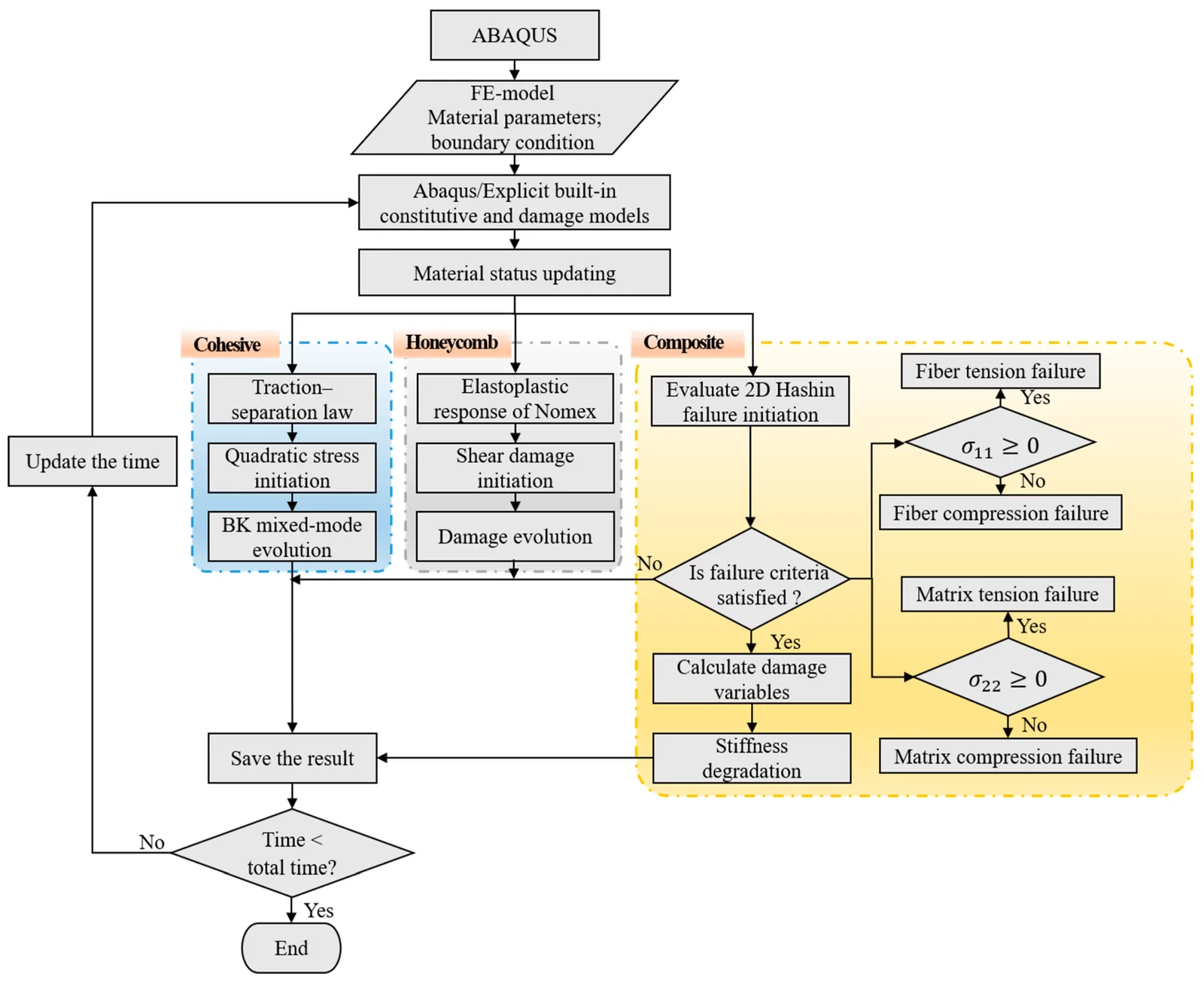
Constitutive and progressive-damage models implemented in Abaqus/Explicit for the cohesive interfaces, explicitly modeled honeycomb cell walls, and composite face sheets.

**Figure 6:**
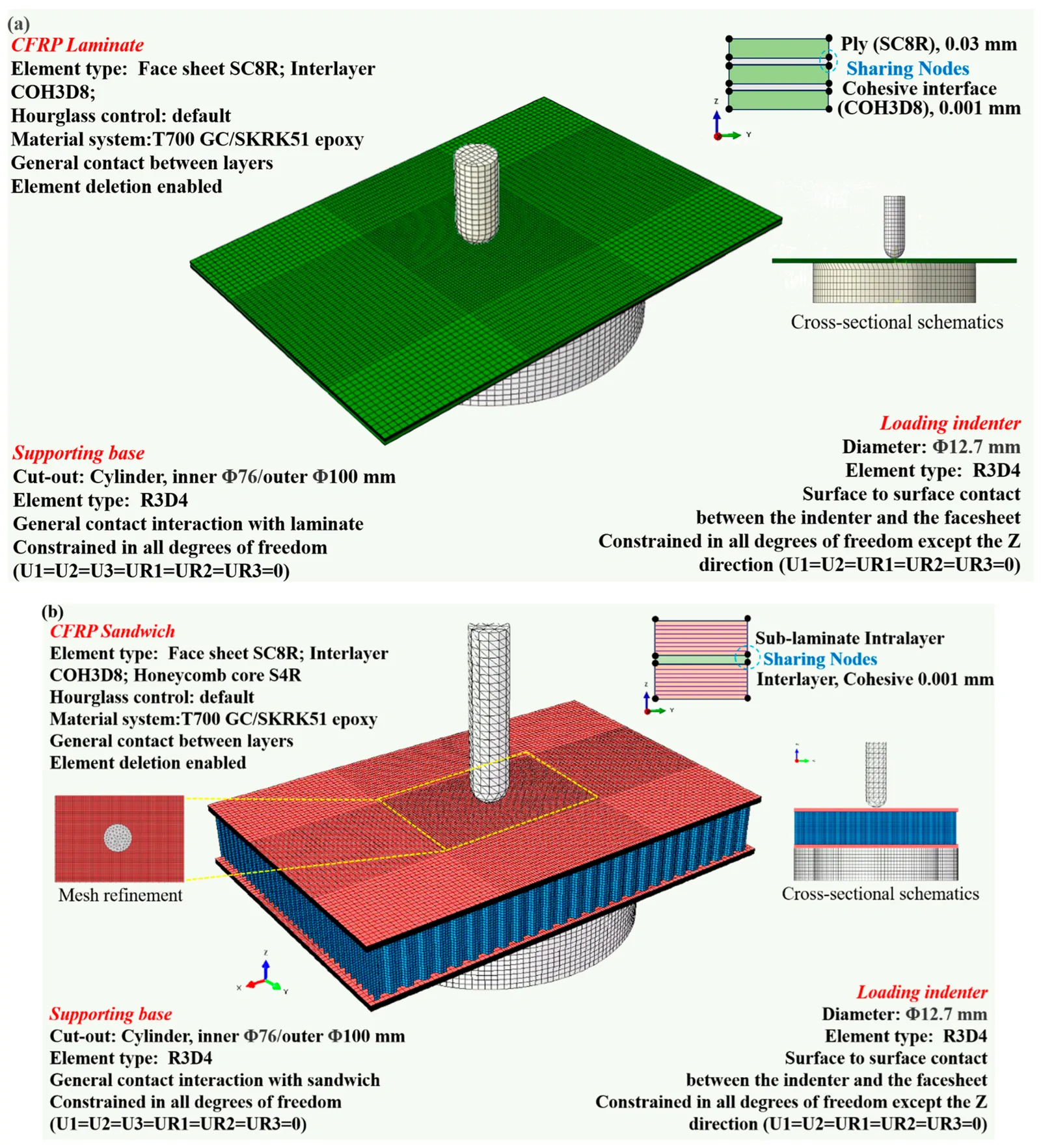
Finite-element models used for quasi-static penetration simulations: (**a**) ply-resolved model of the monolithic laminate and (**b**) sublaminate-based model of the honeycomb sandwich panel.

Finite element analyses were performed in Abaqus/Explicit 2020 to reproduce the quasi-static penetration response and examine the internal damage mechanisms of the monolithic laminates and honeycomb sandwich panels. The progressive-damage procedure is summarized in Figure 5. Two levels of face-sheet representation were adopted. The monolithic laminates were modeled ply by ply to resolve intralaminar damage and ply-level delamination, whereas computational sublaminates were used for the face sheets of the complete sandwich panels. Figure 6 shows the sandwich-panel model, including the indenter, composite face sheets, cohesive interfaces, honeycomb core, and support fixture. The element assignments, discretization schemes, and damage evolution are summarized in Table 3, and the material parameters are listed in Table 4.

For the monolithic laminates, each of the 73 physical plies, with an average thickness of approximately 0.03 mm, was represented by one SC8R continuum-shell layer. COH3D8 cohesive elements were inserted between adjacent plies, resulting in 73 continuum-shell layers and 72 damageable interfaces. The local material orientation of each ply was assigned according to the prescribed cross-ply, quasi-isotropic, or helicoidal stacking sequence. This ply-resolved model followed the SC8R/COH3D8 framework previously applied to helicoidal laminates under transverse loading [27,31,32]. It was used to examine fiber and matrix damage, interlaminar delamination, and their interaction through the laminate thickness.

Direct application of the same ply-resolved representation to the sandwich panels would require 146 continuum-shell layers and 144 cohesive interfaces for the two face sheets alone, in addition to the explicitly modeled honeycomb core and contact components. Such a model would impose a substantial computational cost and make the global response increasingly sensitive to the stiffness and thickness assigned to the numerous cohesive interfaces. A sublaminate representation was therefore adopted for the sandwich-panel simulations. Sublaminate-scale and stacked-shell approaches have previously been used in composite impact and delamination analyses to reduce model size while retaining the physical ply sequence within each computational layer [44,45,46]. Following this approach, each 73-ply face sheet was divided into eight computational sublaminates, referred to as the 8-SL model. Seven sublaminates contained nine adjacent physical plies, and the remaining sublaminate contained ten plies. The original fiber-orientation sequence was retained within the composite section assigned to each SC8R sublaminate. COH3D8 elements were introduced only between adjacent sublaminates, giving seven damageable interfaces in each face sheet. This representation retained the through-thickness variation in fiber orientation while reducing the number of cohesive interfaces and the associated accumulated interfacial compliance. The 8-SL model was intended to predict the global penetration response, dominant intralaminar damage, and major inter-sublaminate separation. It was not intended to resolve delamination at every physical ply interface.

**Table 4:** Material parameters used in the finite element models.

	Property	Value
Ply [30]	Single ply thickness	0.03 mm
	Density	1600 kg/m^3^
	Longitudinal Young’s modulus: $E_{1}$	125.3 GPa
	Transverse Young’s modulus: $E_{2}$	8.4 GPa
	In-plane shear modulus: $G_{12}$	4.8 GPa
	Out of plane shear modulus: $G_{13}$	4.8 GPa
	Out of plane shear modulus: $G_{23}$	3.8 GPa
	In-plane Poisson’s ratio: $v_{12}$	0.32
	Tensile strength (fiber direction): $\sigma_{1t}$	2500 MPa
	Compressive strength (fiber direction): $\sigma_{1c}$	1250 MPa
	Tensile strength (transverse direction): $\sigma_{2t}$	60 MPa
	Compressive strength (transverse direction): $\sigma_{2c}$	140 MPa
	In-plane shear strength: $\tau_{12}$	88 MPa
Interface cohesive element [46]	Normal strength: $t_{n}^{0}$	60 MPa
	Shear strength: $t_{s}^{0}$, $t_{t}^{0}$	90 MPa
	Mode-I toughness: $G_{nc}$	0.28 kJ/m^2^
	Mode-II toughness: $G_{sc}$, $G_{tc}$	0.79 kJ/m^2^
	B–K law parameter: *η*	1.5
Honeycomb Core	Cell side length	1.83 mm
	Density	128 kg/m^3^
	Core height	20 mm
	Out-of-plane compressive strength	10 MPa

Intralaminar damage in both the ply-resolved and sublaminate models was described using the two-dimensional Hashin criterion. Fiber tension, fiber compression, matrix tension, and matrix compression were evaluated at the material integration points of the SC8R elements. Once a damage-initiation criterion was satisfied, the corresponding stiffness components were progressively degraded according to an energy-based evolution law. Severely degraded elements were deleted to permit complete penetration and to limit excessive mesh distortion.

The COH3D8 elements followed a traction-separation law. Delamination initiation was governed by a quadratic nominal-stress criterion, while mixed-mode damage propagation was described using the Benzeggagh–Kenane energy criterion. Damage in the SC8R elements therefore represents intralaminar fiber or matrix failure, whereas damage in the COH3D8 elements represents interlaminar separation. This distinction was used when interpreting the numerical damage distributions in Section 3. The hexagonal Nomex honeycomb core was explicitly represented using S4R shell elements to reproduce cell-wall folding, crushing, and local fracture. The Nomex paper was described by an elastoplastic constitutive model with shear-damage initiation and evolution. The shear-damage initiation criterion is based on the equivalent plastic strain at damage initiation as a function of the shear stress ratio and equivalent plastic strain rate. The corresponding Abaqus output variable SHRCRT was used to identify the onset of shear-related failure in the explicitly modeled honeycomb cell walls, with SHRCRT = 1 indicating that the initiation criterion was satisfied.

The upper and lower face sheets were connected to the honeycomb core using tie constraints, representing ideal face-core bonding. Face-core debonding was therefore not included as an independent failure mode in the numerical model. The hemispherical indenter and cylindrical support were modeled as rigid bodies using R3D4 elements. General contact with hard normal behavior and a tangential penalty friction coefficient of 0.2 was defined between the rigid and deformable components and among deformable surfaces following material failure. The support was constrained in all translational and rotational degrees of freedom. The indenter was restricted to vertical translation and driven by a prescribed displacement. The support opening diameter, indenter position, and loading direction were consistent with the experimental configuration.

As shown in Figure 6, the mesh was refined beneath the indenter and within the expected penetration region. A small viscosity coefficient of $1\times 10^{-7}$ was assigned to the four Hashin damage modes to regularize the progressive stiffness degradation and improve numerical stability, consistent with previous Hashin-based CFRP simulations [47,48,49]. Element deletion was enabled, with the maximum degradation parameter set to 0.99. The internal, kinetic, and viscous dissipated energies were monitored to assess the quasi-static character and numerical stability of the Abaqus/Explicit simulations. The ratio of kinetic energy to internal energy remained below 5% throughout most of the loading process, indicating that inertial effects were sufficiently small for the response to be regarded as quasi-static. In addition, the viscous dissipated energy remained much smaller than the internal energy throughout the analysis, confirming that the numerical dissipation introduced by the viscous regularization was minor.

## 3. Results and Discussion

### 3.1. Experimental Response of Monolithic Laminates

**Figure 7:**
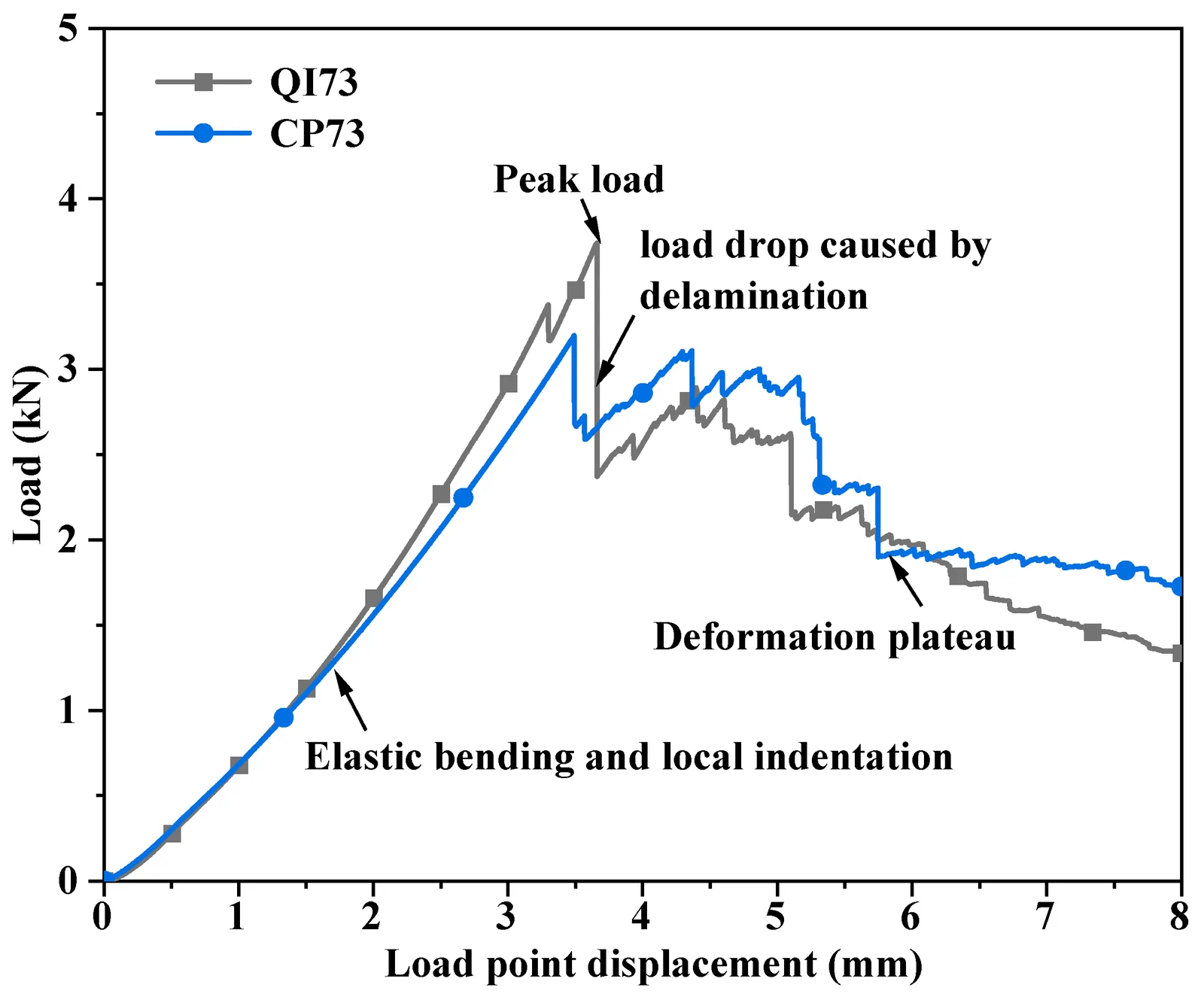
Representative load–displacement response of 73-ply laminate specimens under quasi-static penetration, showing the characteristic stages of elastic bending, peak load, damage-associated load drop, and post-peak deformation plateau.

The out-of-plane load–displacement responses of the monolithic laminates were first examined to evaluate their load-bearing capacity and energy absorption under quasi-static penetration. As illustrated in Figure 7, using CP73 and QI73 as representative baseline configurations, the penetration response can generally be divided into four characteristic stages: (I) an initial elastic bending/indentation stage, (II) a nonlinear damage-accumulation stage leading to the peak load, (III) damage-induced load drops associated with rapid crack propagation and delamination growth, and (IV) a post-peak deformation plateau after major structural damage. In the initial stage, the load increased approximately linearly with the load-point displacement, indicating that the laminate response was mainly governed by elastic bending and local indentation beneath the hemispherical indenter. With increasing displacement, the response gradually became nonlinear as intralaminar damage and interlaminar separation began to accumulate. The peak load corresponds to the maximum load-bearing capacity of the laminate before dominant damage coalescence. After the peak load, abrupt load drops were observed, indicating rapid development and coalescence of internal damage. The post-test observations and numerical results presented later in Section 3.2 show that both intralaminar damage and interlaminar delamination were involved in this failure process. Previous studies [27,31,50] on helicoidal laminates under transverse loading have further shown that sudden load reductions can occur when cracks propagating from the tensile surface interact with delaminated interfaces. Such crack–delamination interaction therefore provides a possible explanation for the abrupt load drops observed here, although the present study does not directly resolve this event sequence in time. In the final stage, the fractured laminate still carried residual load through fiber bridging, frictional contact, and continued deformation around the penetrated region, resulting in a post-peak deformation plateau. In this study, failure was defined as a sudden reduction in the applied load exceeding 30% of the peak load. The penetration energy was then calculated as the area under the load–displacement curve up to this critical failure point.

**Figure 8:**
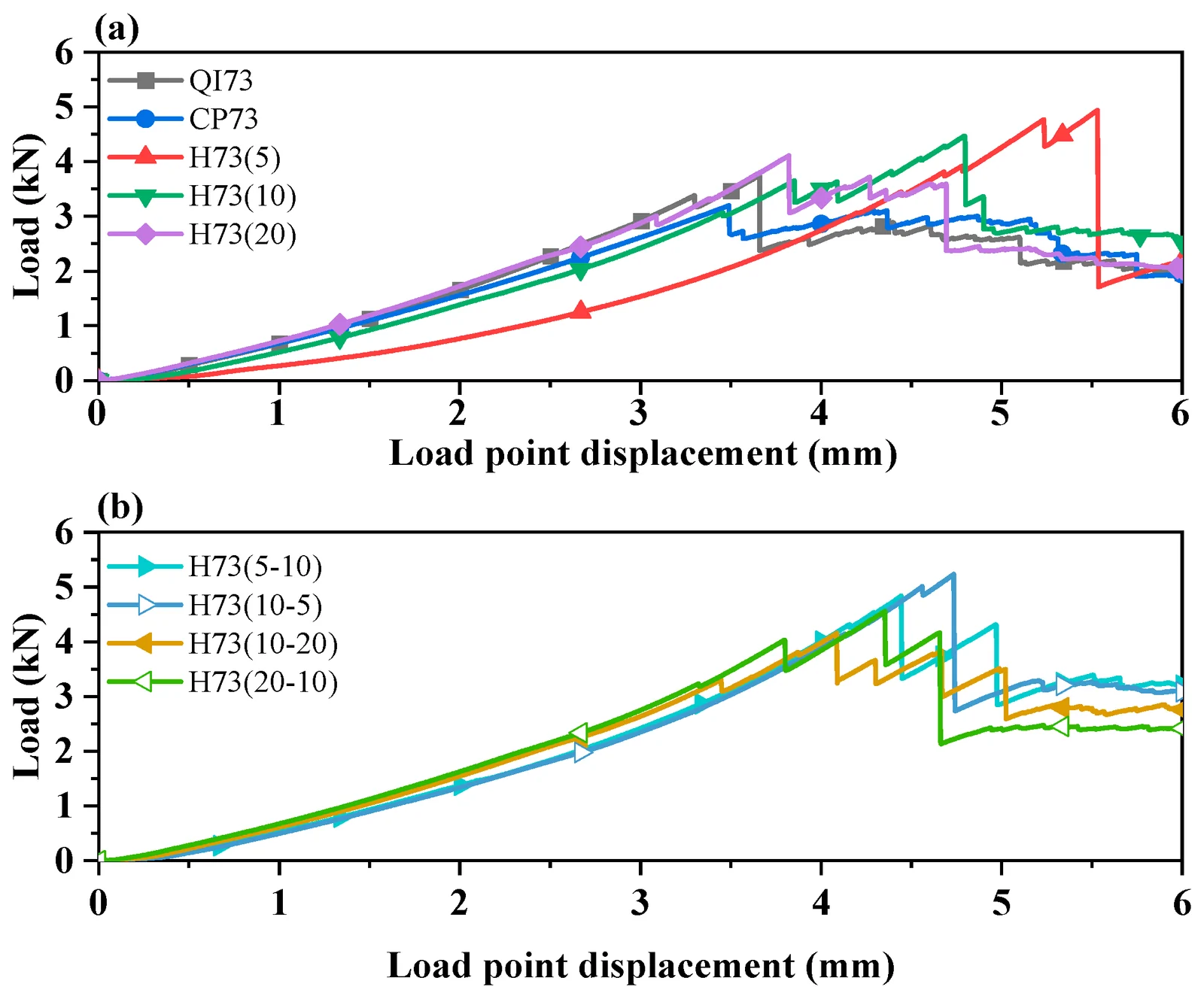
Load–displacement responses of the 73-ply laminate specimens under quasi-static penetration: (**a**) uniform helicoidal laminates compared with QI73 and CP73; and (**b**) hybrid helicoidal laminates with different through-thickness distributions of the inter-ply angle.

The complete load–displacement curves of the 73-ply laminates are shown in Figure 8, and the corresponding quantitative results are summarized in Table 5. Among the laminates with uniform inter-ply angles (Figure 8a), H73(5) achieved the highest peak load of 4.94 kN, followed by H73(10) with 4.29 kN. Both values were higher than those of QI73 and CP73, which reached peak loads of 3.86 kN and 3.58 kN, respectively. This confirms that small-angle helicoidal layups can improve the transverse load-bearing capacity of thin-ply laminates. However, H73(20) showed a reduced peak load of 3.66 kN, close to that of the conventional reference laminates. Therefore, increasing the inter-ply angle beyond an appropriate range does not necessarily lead to further improvement. The penetration energy showed a slightly different trend from the peak load. H73(10) exhibited the highest penetration energy among the uniform helicoidal laminates, reaching 10.55 J, whereas H73(5) reached 9.75 J and H73(20) reached 9.23 J. This indicates that the energy absorption capacity is not governed solely by the maximum load. Instead, it depends on the combined effects of peak load, failure displacement, and post-peak residual load-carrying behavior. Therefore, H73(5) exhibited the highest peak load and peak displacement among the uniform helicoidal laminates, whereas H73(10) sustained a larger integrated load over the pre-failure response and consequently achieved the highest penetration energy. Mass normalization further differentiates the relative energy-absorption performance among the tested laminate configurations. H73(5) exhibited the highest SEA of 0.197 kJ/kg, followed by H73(10) at 0.190 kJ/kg, although H73(10) showed the highest absolute penetration energy among the uniform helicoidal laminates. The SEA of H73(5) was approximately 14.8% and 54.2% higher than those of CP73 and QI73, respectively. These results show that, within the present test configuration, the layup maximizing peak load or absolute penetration energy does not necessarily maximize the specimen-level mass-normalized energy absorption. The energy-absorption efficiency showed a different ranking, reflecting the ability of each configuration to sustain load relative to its own peak value rather than its absolute energy capacity.

Motivated by the through-thickness variation in pitch observed in natural Bouligand structures, non-uniform helicoidal layups were also investigated (Figure 8b). For the non-uniform helicoidal laminates, the through-thickness arrangement of inter-ply angles had a clear influence on the penetration response. H73(10–5), which had the larger 10° inter-ply angle in the lower half and the smaller 5° inter-ply angle in the upper half, exhibited the highest peak load among all laminate configurations, reaching 5.23 kN. This value was 35.5% higher than that of QI73 and 46.1% higher than that of CP73. H73(10–5) also outperformed its reversed counterpart H73(5–10), which reached a peak load of 4.84 kN. A similar but less pronounced trend was observed between H73(20–10) and H73(10–20), with H73(20–10) showing a slightly higher peak load.

This result suggests that placing a relatively larger inter-ply angle near the lower tensile side and a smaller inter-ply angle near the upper loading side is beneficial for improving penetration resistance. Smaller inter-ply angles tend to improve delamination resistance, whereas larger inter-ply angles are more effective in resisting matrix splitting. Since delamination is more likely to initiate near the upper half under local transverse loading, and matrix splitting tends to develop from the lower tensile surface, the H73(10–5) configuration provides a more balanced resistance to both damage modes.

**Figure 9:**
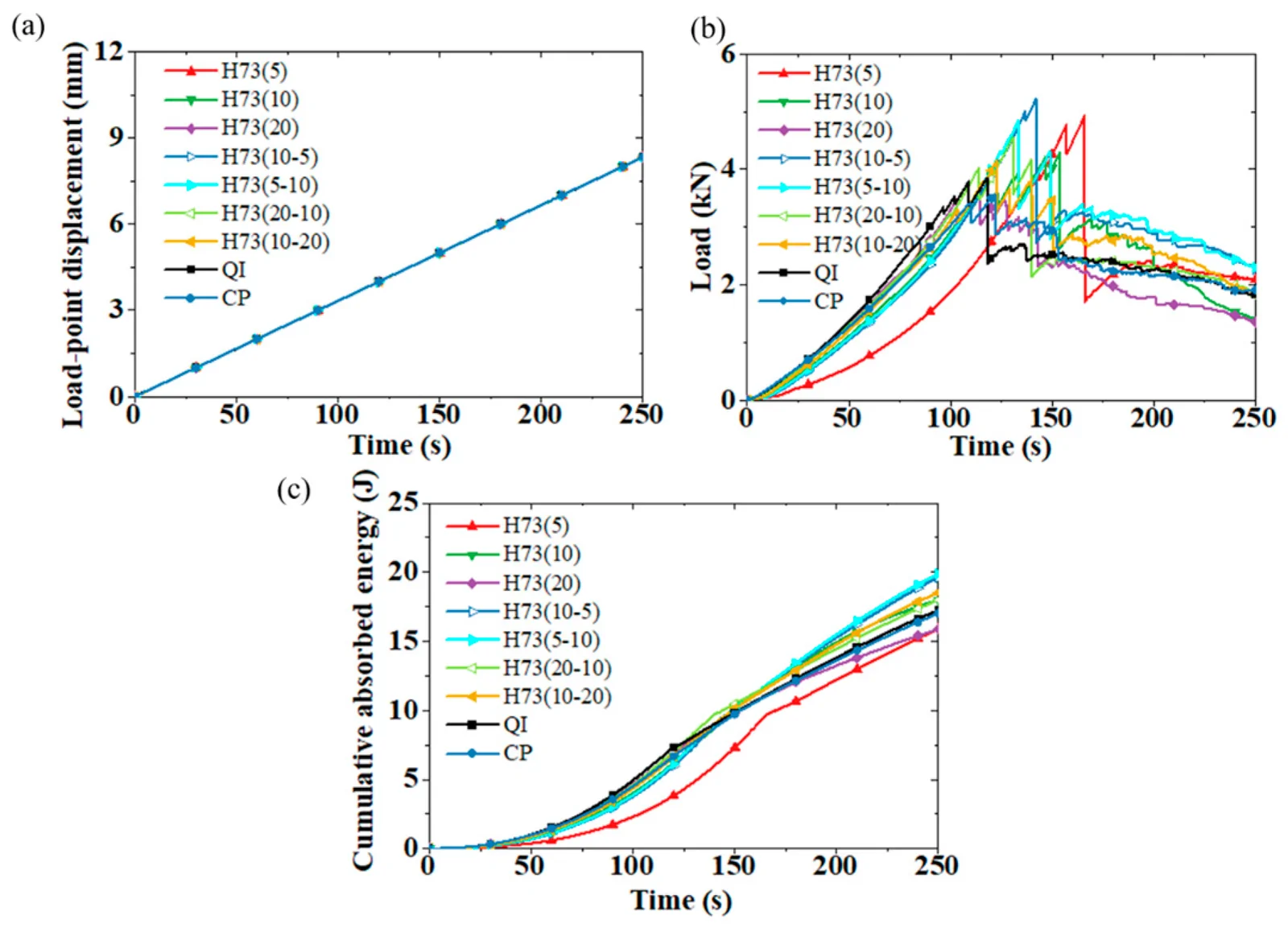
Time histories of the 73-ply laminate specimens during quasi-static penetration: (**a**) load-point displacement, where the curves overlap because all specimens were tested at the same constant crosshead speed of 2.0 mm/min, (**b**) applied load, and (**c**) cumulative absorbed energy obtained by integrating the load with respect to displacement.

To complement the load–displacement responses, Figure 9 presents the corresponding time histories of load-point displacement, applied load, and cumulative absorbed energy. Under the prescribed quasi-static loading, the displacement increases continuously with time, whereas the load histories exhibit distinct peaks and subsequent drops as damage develops. The cumulative-energy curves increase progressively throughout loading and continue to rise after the peak load, indicating continued energy dissipation during post-peak damage and deformation. The differences among the layups therefore arise not only from their peak load but also from the manner in which load carrying and energy absorption are sustained during progressive penetration. The cumulative-energy histories in Figure 9 describe the complete recorded loading process, whereas the penetration-energy values reported in the tables are evaluated up to the predefined critical failure point.

The initial stiffness, $K_{0}$, was obtained by least-squares fitting of the approximately linear portion of each load–displacement curve. The fitted values and regression statistics are listed in Table 6. The high correlation coefficients indicate that the selected intervals were adequately represented by linear regression. QI73 exhibited an initial stiffness of 1068 N/mm, while CP73 had a lower value of 931 N/mm. Among the uniform helicoidal laminates, H73(5) showed the lowest stiffness of 864 N/mm, consistent with its greater early-stage compliance. The values increased to 975 N/mm for H73(10) and 988 N/mm for H73(20).

The hybrid laminates generally showed higher initial stiffnesses. H73(10–5) reached the highest value of 1078 N/mm, slightly above that of QI73, and also exhibited the highest peak load. Thus, its load-bearing advantage was accompanied by a relatively stiff initial response rather than by increased compliance. Nevertheless, $K_{0}$ should be considered together with the peak load, failure displacement, penetration energy, and observed damage, since stiffness alone does not describe penetration resistance.

**Figure 10:**
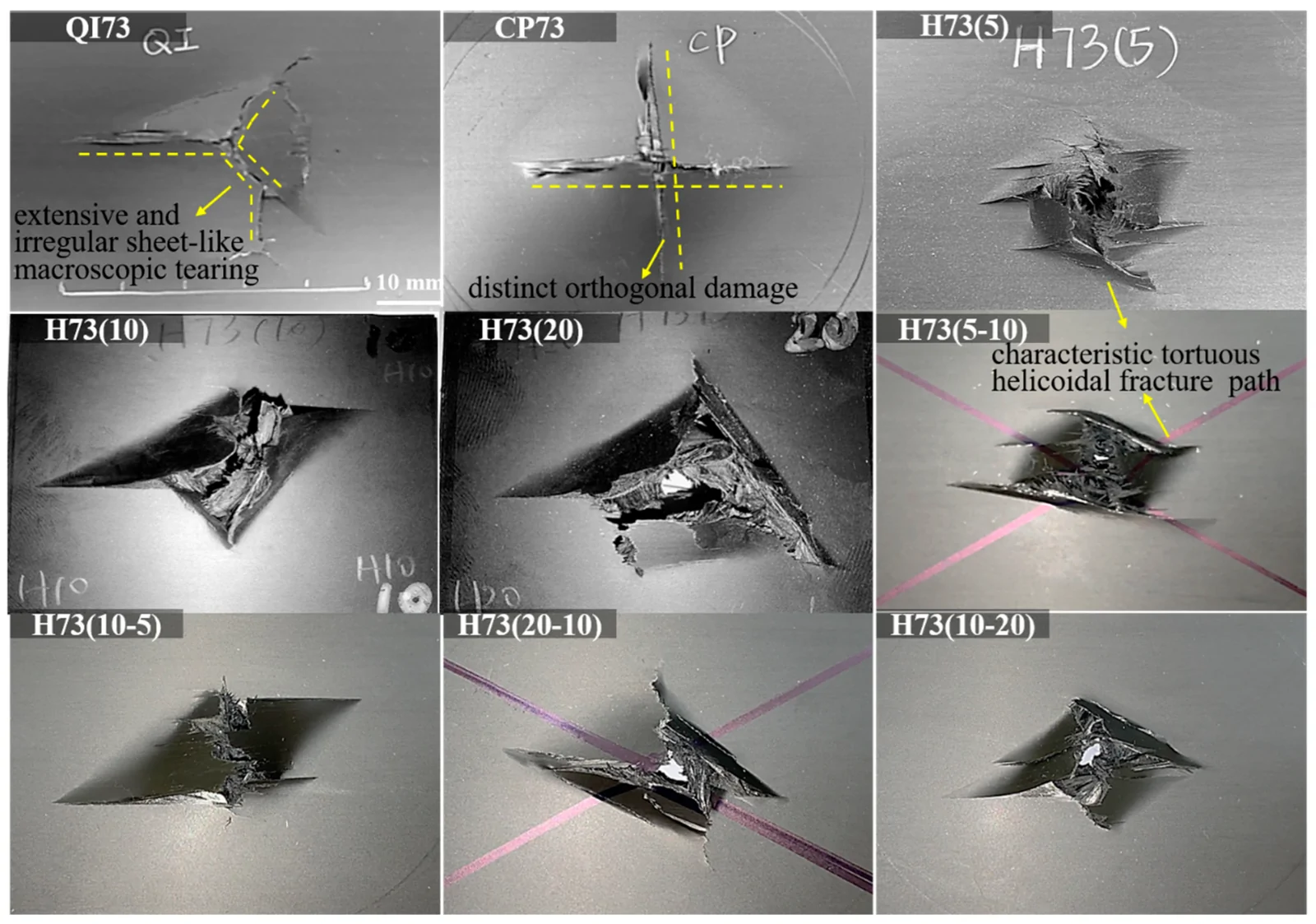
Back-face damage morphologies of the 73-ply laminate specimens after quasi-static penetration. CP73 exhibits orthogonal splitting along the principal fiber directions, whereas the helicoidal laminates show layup-dependent crack deflection, spiral-like tearing, and flap formation.

The back-face morphologies in Figure 10 reveal pronounced layup-dependent failure patterns. The conventional CP73 laminate failed with a distinct orthogonal crack pattern, with cracks aligned primarily along the 0° and 90° fiber directions. This indicates that damage propagation in the cross-ply laminate was strongly constrained by the two dominant fiber orientations. In contrast, the QI73 laminate showed a more irregular and asymmetric fractured region, suggesting that the crack path was redistributed among multiple fiber directions rather than being confined to two orthogonal directions. The helicoidal laminates displayed markedly different damage features. For H73(5) and H73(10), the damaged region exhibited a clear twisting or spiral-like morphology instead of the orthogonal crack pattern observed in CP73. This suggests that the gradual rotation of fiber orientation through the thickness deflected the propagating cracks and prevented the formation of a single straight through-thickness fracture path. Such a spiraling damage morphology is consistent with previous observations on helicoidal laminates under transverse loading, where delamination and matrix cracking were shown to climb progressively through the laminate thickness rather than remaining confined to a small number of fixed ply directions. When the inter-ply angle increased to 20°, the damage became more localized and was accompanied by more pronounced sheet-like tearing. This indicates that an excessively large inter-ply mismatch may weaken the ability of the helicoidal architecture to guide damage gradually through the thickness, thereby promoting more localized fracture. Similar trends were observed in the non-uniform helicoidal laminates. In particular, H73(10–5) exhibited a relatively compact but well-developed damage region, whereas H73(5–10) and H73(20–10) showed more extended matrix splitting or flap-like damage on the back face. These observations indicate that the penetration damage mode is governed not only by the magnitude of the inter-ply angle but also by its through-thickness distribution.

The macroscopic damage patterns provide an initial explanation for the superior mechanical response of the small-angle and hybrid helicoidal laminates. In small-angle helicoidal laminates, crack propagation is continuously redirected by the incrementally rotating plies, forcing the damage to evolve along a more tortuous path. This crack deflection mechanism can delay through-thickness damage coalescence and contribute to higher penetration resistance. For the non-uniform H73(10–5) configuration, the combination of a smaller inter-ply angle near the upper loading side and a larger inter-ply angle near the lower tensile side appears to provide a favorable balance between delamination resistance and resistance to matrix splitting. However, the competition between intralaminar matrix cracking, fiber breakage, and interlaminar delamination cannot be fully resolved from post-mortem surface observations alone. Therefore, finite element simulations are further used in the following section to reveal the internal damage evolution and clarify the mechanisms responsible for the observed differences among layup configurations.

**Figure 11:**
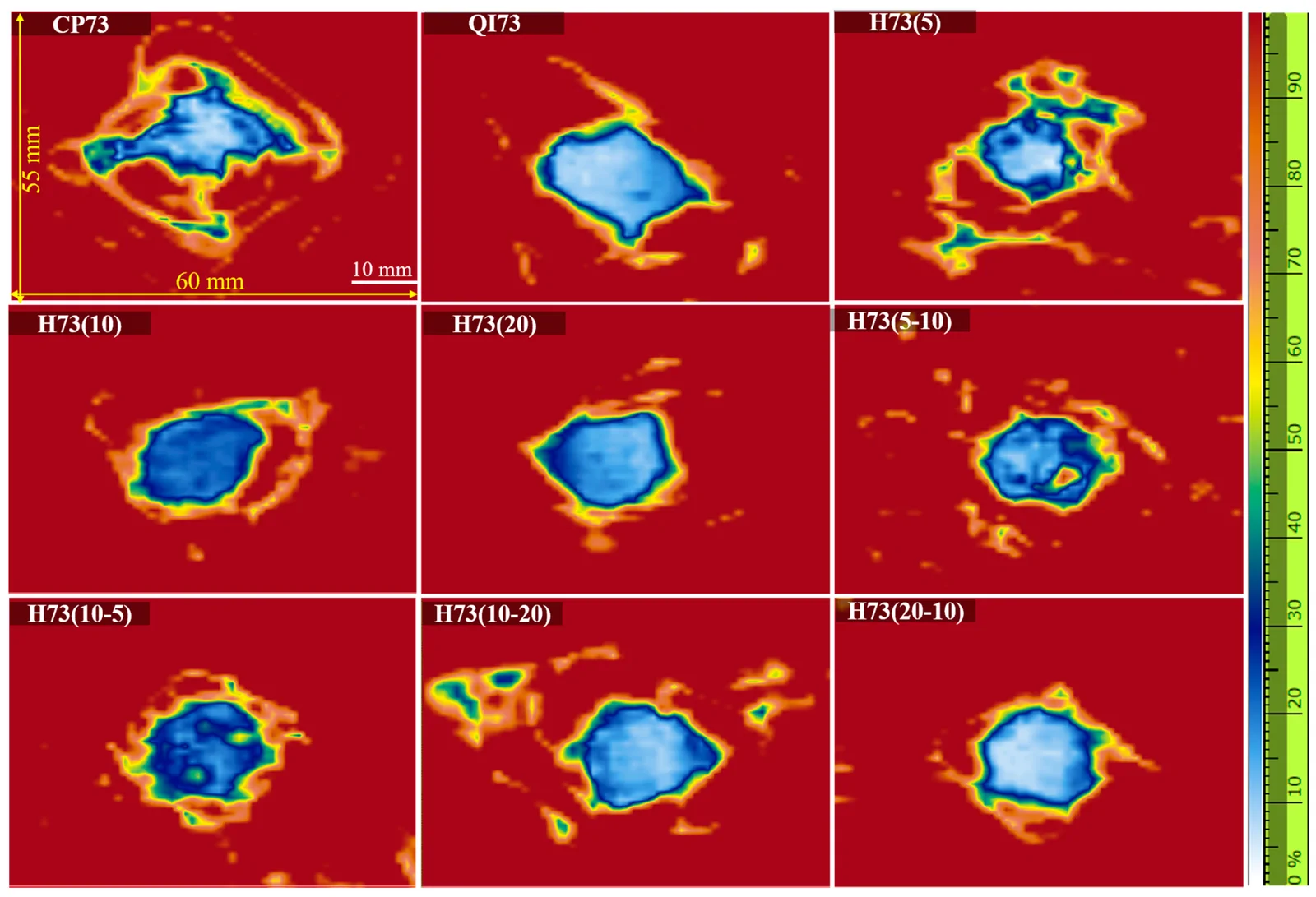
Ultrasonic C-scan maps of the 73-ply laminate specimens after quasi-static penetration. The central indications correspond to the penetrated regions, while the surrounding contrast variations show the projected extent of subsurface damage. All maps are presented using the same spatial and color scales.

Ultrasonic C-scan maps were therefore used to examine the projected internal damage, as shown in Figure 11. The central signal-loss regions correspond to the penetrated zones, while the surrounding contrast contours indicate subsurface damage extending beyond the visible openings. CP73 exhibited a broad, lobed damage pattern with directional extensions that were consistent with its orthogonal back-face cracking. The QI73 indication was more compact, although an elongated branch remained visible along one direction. The helicoidal laminates exhibited distinct changes in both the extent and spatial distribution of the C-scan indications. H73(5) produced a broad and irregular damage field with several peripheral branches, consistent with distributed crack deflection through the rotating plies. The indications became more compact for H73(10) and H73(20), particularly for H73(20), whose projected damage was concentrated near the penetration region. The hybrid laminates also showed a clear sequence dependence. H73(10–5) retained a compact central damage region surrounded by a relatively continuous irregular boundary, whereas H73(5–10) contained more scattered peripheral indications. H73(10–20) showed several detached damage indications away from the central region, while the projected damage of H73(20–10) remained comparatively confined.

The C-scan maps do not distinguish individual matrix cracks, fiber fractures, and delaminated interfaces through the thickness. They nevertheless confirm that both the inter-ply angle and its through-thickness sequence influence the projected internal damage field. Together with the load–displacement results and the back-face photographs, these observations show that the improved performance of H73(10–5) resulted from a favorable combination of high load capacity and controlled damage development. The internal progression of the different damage modes is examined numerically in Section 3.2.

### 3.2. Numerical Interpretation of Laminate Damage Mechanisms

**Figure 12:**
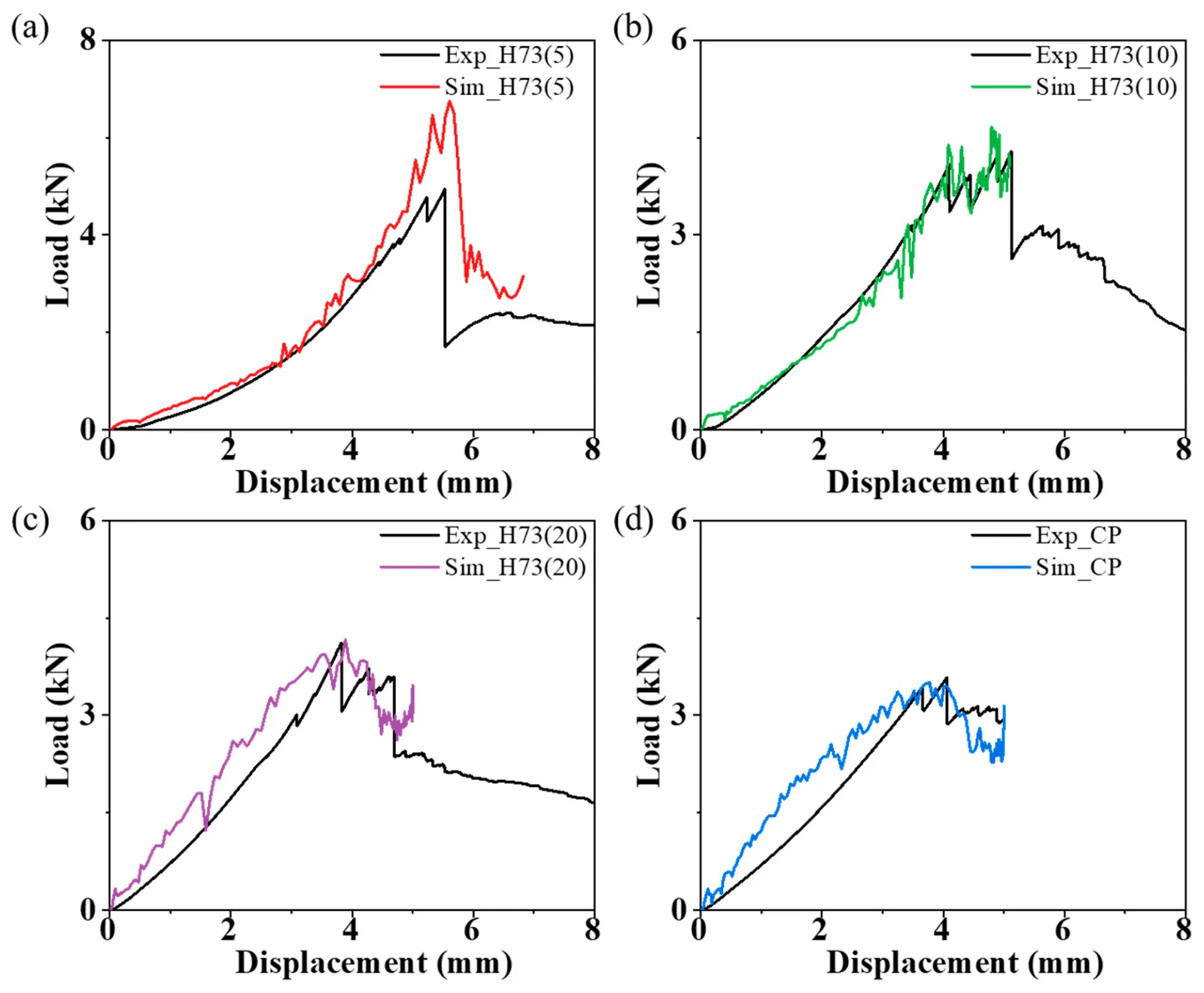
Comparison of experimental and numerical load–displacement responses for representative 73-ply monolithic laminates: (**a**) H73(5), (**b**) H73(10), (**c**) H73(20), and (**d**) CP73.

The ply-resolved laminate model was further assessed by comparing the experimental and numerical simulation load–displacement responses of representative configurations, as shown in Figure 12. CP73 and the uniform helicoidal laminates H73(5), H73(10), and H73(20) were selected to span the principal range of stacking architectures considered in the study. Without layup-specific parameter adjustment, the model reproduced the overall loading trend, peak-load range, and principal post-peak response of the tested laminates. Local discrepancies remained in the exact peak magnitude and abrupt post-peak fluctuations, reflecting the difficulty of reproducing individual crack-coalescence and delamination events point by point. Overall, the agreement was considered sufficient for the subsequent mechanism-oriented interpretation of intralaminar damage and interlaminar separation.

**Figure 13:**
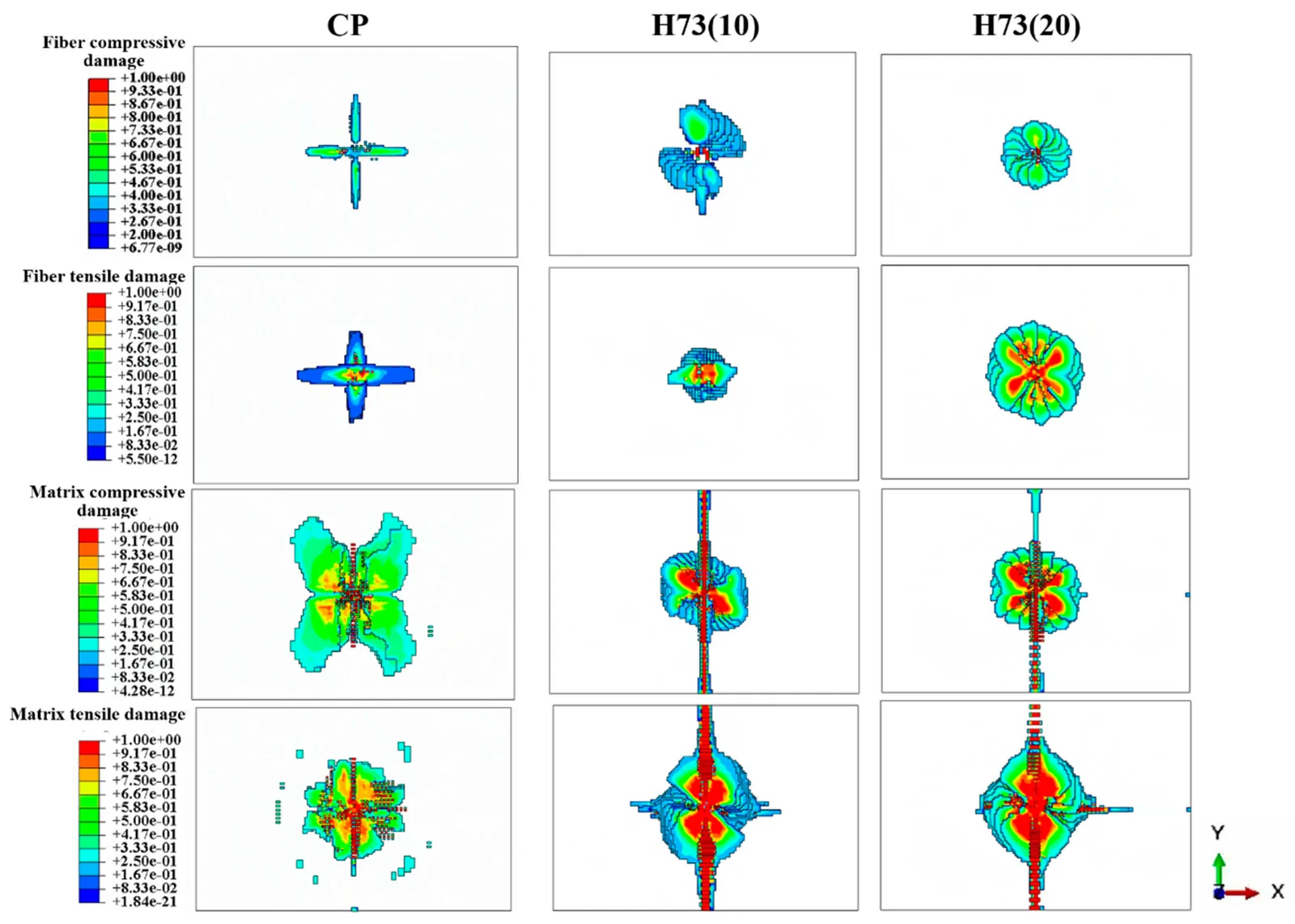
Through-thickness projections of the numerically predicted intralaminar damage in CP73, H73(10), and H73(20) at their respective failure states. The columns correspond to the three laminate configurations, while the rows show fiber-compression, fiber-tension, matrix-compression, and matrix-tension damage.

The back-face photographs in Figure 10 and the ultrasonic C-scan maps in Figure 11 demonstrate that the laminate architecture strongly affected both the external fracture morphology and the projected internal damage field. To identify the intralaminar origin of these differences, Figure 13 compares the predicted fiber- and matrix-damage distributions of CP73, H73(10), and H73(20) at their corresponding failure stages. These contours represent the local stiffness degradation predicted by the Hashin model and should therefore be interpreted as distributed damage zones rather than discrete crack surfaces. For CP73, all four damage modes retained a strong directional dependence. Fiber-tensile and fiber-compressive damage extended predominantly along the two principal material directions, while matrix-tensile damage formed orthogonal bands around the indentation region. Matrix-compressive damage was distributed over a broader four-lobed region but remained symmetric with respect to the 0° and 90° axes. Because the same two fiber orientations are repeated through the laminate thickness, damaged regions in adjacent plies tend to overlap along the same directions. This through-thickness alignment provides preferential paths for crack coalescence and accounts for the cross-shaped splitting observed on the back face of CP73 in Figure 10.

A different damage organization was obtained for H73(10). The material axes rotate by 10° between adjacent plies, and the preferred directions of fiber and matrix damage consequently change through the thickness. When projected onto the laminate plane, the damaged regions form overlapping fan-shaped and rotating sectors rather than two fixed orthogonal bands. Matrix damage is distributed among several orientations around the indentation zone, while fiber damage is less strongly confined to a single pair of directions. A crack segment initiated in one ply therefore cannot continue along an unchanged plane through the entire thickness but must be redirected as it enters plies with different fiber orientations. The accumulated rotation of these damaged sectors provides the numerical basis for the twisted and spiral-like back-face morphology observed experimentally.

The more distributed and non-collinear damage in H73(10) also helps explain its relatively high penetration energy. Damage develops over several orientations before a continuous failure path is established, allowing the laminate to maintain load while deformation and matrix-dominated damage accumulate. This behavior differs from the rapid alignment and coalescence of the damage bands in CP73. It is consistent with the experimental result that H73(10) exhibited the highest penetration energy among the uniform helicoidal laminates, although its peak load was lower than that of H73(5). Increasing the inter-ply angle to 20° changed the damage field again. In H73(20), the projected damage formed more distinct rosette-like lobes, accompanied by pronounced fiber-tensile and fiber-compressive damage in several broad sectors. The larger change in material orientation between adjacent plies reduces the continuity of the gradual damage rotation. Instead of forming a smoothly evolving helicoidal path, damage tends to coalesce within larger groups of plies and produces wider damaged sectors. Once these sectors become connected, several plies can separate and deform together, leading to the sheet-like or flap-like tearing observed on the back face of H73(20).

The comparison in Figure 13 therefore indicates that the macroscopic fracture morphology is controlled not simply by the total amount of damage but by its directional coherence through the laminate thickness. Repeated 0°/90° orientations in CP73 produce aligned damage bands and orthogonal splitting. The intermediate 10° rotation progressively redirects the damage and produces a twisted fracture path. At 20°, the larger angular mismatch interrupts this gradual redirection and favors the formation of broader damaged sectors and ply-block tearing. These numerical results reproduce the principal differences observed in the back-face photographs and support the experimentally identified dependence of penetration resistance on the inter-ply angle.

**Figure 14:**
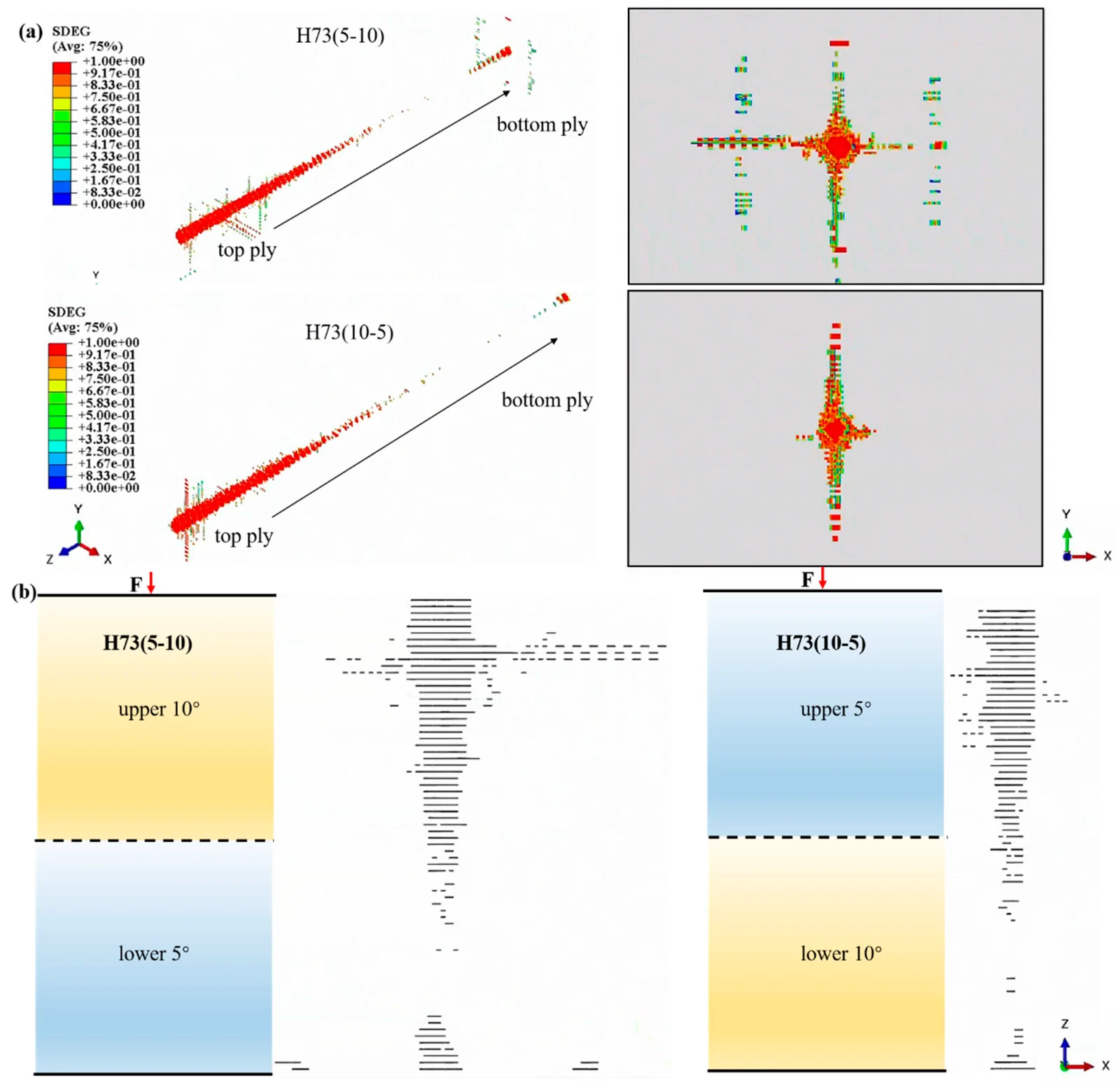
Predicted interlaminar damage in H73(5–10) and H73(10–5) at their respective peak-load states: (**a**) isometric views and in-plane projections of the cohesive damage variable SDEG; and (**b**) through-thickness distributions of damaged cohesive elements and the corresponding inter-ply-angle arrangements in the upper and lower halves of the laminates.

The preceding results showed that reversing the through-thickness angle distribution altered the penetration response of the hybrid helicoidal laminates. To clarify this sequence effect, Figure 14 compares the cohesive-interface damage in H73(10–5) and H73(5–10). Following the notation adopted in this study, the first angle refers to the lower tensile-side half of the laminate, while the second refers to the upper loading-side half. The cohesive damage variable SDEG ranges from zero for an undamaged interface to unity for complete interface degradation.

As shown in Figure 14, the two laminates developed different interlaminar-damage patterns around the indentation region. In H73(10–5), the projected cohesive damage remained relatively concentrated near the penetration zone, with limited extension along the principal in-plane directions. By contrast, H73(5–10) developed longer lateral branches and a more pronounced cross-shaped projection. The through-thickness views further indicate that the angle transition affected the position and continuity of the damaged interfaces, rather than simply changing the total amount of delamination. This difference can be related to the asymmetric stress state through the laminate thickness. In H73(10–5), the smaller 5° inter-ply angle in the upper half reduces the orientation mismatch between adjacent plies near the loading side, thereby limiting the lateral growth and coalescence of local delamination. Reversing the sequence places the larger 10° angle in this region and produces a more directionally extended interlaminar-damage field. Isolated interface damage close to the support boundary was not included in this interpretation because it was induced by the local support condition rather than by the central penetration process.

**Figure 15:**
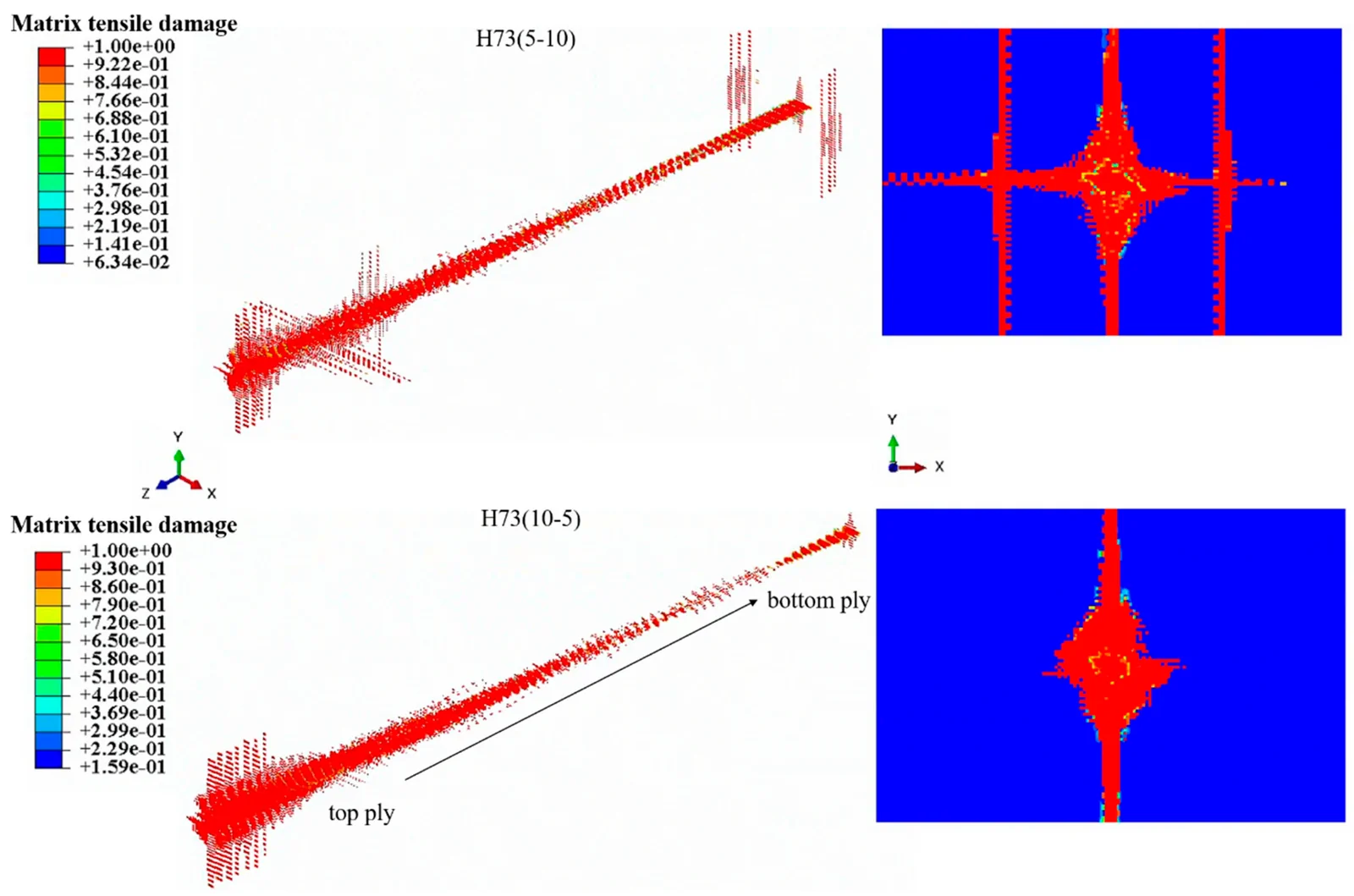
Predicted matrix tensile damage in H73(5–10) and H73(10–5) at their respective peak-load states. Isometric views and back-face projections show the spatial development of the matrix-tensile damage variable.

The corresponding matrix-tensile damage is shown in Figure 15. H73(5–10) exhibited long, cross-shaped matrix-damage bands extending from the central penetration zone, whereas the damage in H73(10–5) was more confined around the indenter and showed substantially less lateral splitting. Matrix cracks initiate primarily from the lower surface subjected to bending-induced tension. The larger 10° inter-ply angle in the lower half of H73(10–5) changes the preferred cracking direction more rapidly between adjacent plies and interrupts the development of a long, continuous matrix split. In H73(5–10), the smaller 5° angle in the lower half permits the tensile-side cracks to remain approximately aligned over a greater thickness and distance, resulting in more extensive directional splitting.

The higher peak load of H73(10–5) can therefore be attributed to the combined action of the two angle regions. The smaller angle near the loading side restricts the development of extended delamination, while the larger angle near the tensile side limits the continuity of matrix splitting. The reverse sequence does not provide the same spatial correspondence between the local damage mode and the inter-ply angle. Consequently, interlaminar and intralaminar damage connect earlier in H73(5–10), leading to a lower peak load and penetration energy. The H73(20–10) and H73(10–20) pair exhibited the same ordering in peak load, and its detailed damage contours are not repeated here.

### 3.3. Mechanical Response of Sandwich Panels

#### 3.3.1. Characteristic Two-Peak Penetration Response

**Figure 16:**
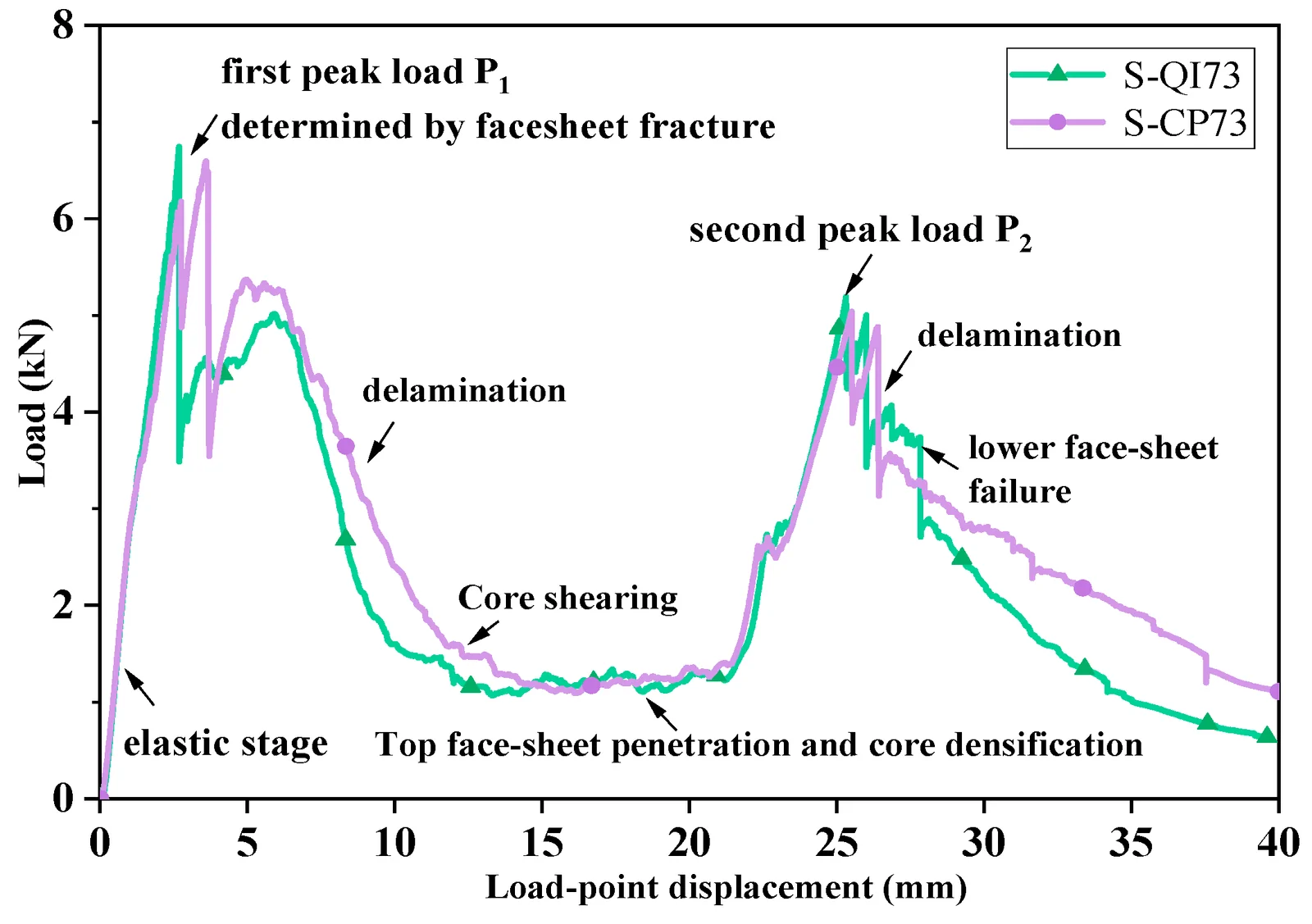
Representative load–displacement response of honeycomb sandwich panels under quasi-static penetration, showing the sequential stages of upper-face-sheet loading and perforation, honeycomb-core crushing and densification, lower-face-sheet loading and failure, and final penetration.

The introduction of the thick honeycomb core fundamentally alters the out-of-plane penetration behavior of the composite structures. Figure 16 shows a representative load–displacement response of the honeycomb sandwich panels under quasi-static penetration. Compared with the monolithic laminates, the sandwich panels exhibited a more complex multi-stage response owing to the sequential interaction among the upper face sheet, honeycomb core, and lower face sheet. The response can be divided into five characteristic regimes: initial elastic indentation, first peak load $P_{1}$, core-crushing plateau, reloading toward the second peak load $P_{2}$, and final post-penetration softening. In the initial stage, the load increased almost linearly with the load-point displacement. This stage was governed by local indentation of the upper face sheet, bending of the sandwich panel, and elastic support from the honeycomb core. The first peak load $P_{1}$ occurred when the upper face sheet reached its local load-bearing limit and underwent perforation around the indenter. The subsequent sudden load drop was associated with local upper-face damage, interlaminar delamination within the face sheet, and the onset of core crushing beneath the indenter. After this drop, the load entered a relatively low and stable plateau, corresponding mainly to progressive crushing and densification of the honeycomb core. As the indenter continued to move downward, the load increased again when the compressed core and indenter began to transfer load to the lower face sheet. The second peak load $P_{2}$ was reached when the lower face sheet failed under bending-dominated tensile loading. After $P_{2}$, the residual load gradually decreased as the lower face sheet fractured and the crushed core continued to deform.

**Figure 17:**
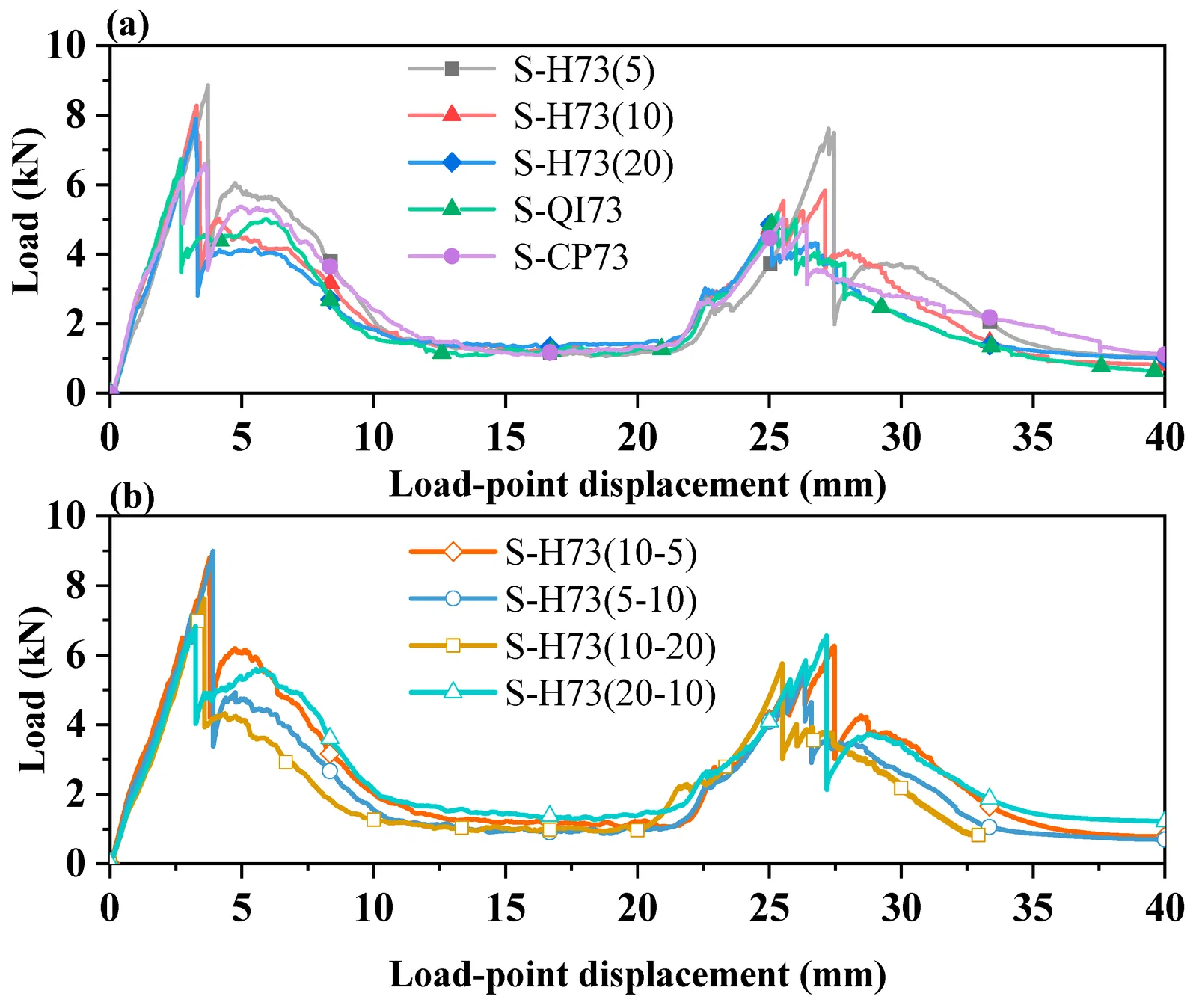
Load–displacement responses of honeycomb sandwich panels with 73-ply face sheets under quasi-static penetration: (**a**) panels with uniform helicoidal face-sheet layups compared with S-QI73 and S-CP73; and (**b**) panels with non-uniform helicoidal face-sheet layups.

The complete load–displacement curves of the tested sandwich panels are shown in Figure 17, and the corresponding quantitative results are summarized in Table 7. The conventional S-QI73 and S-CP73 panels reached first peak loads of 6.74 kN and 6.59 kN, respectively, and second peak loads of 5.19 kN and 5.04 kN. In comparison, the sandwich panels with helicoidal face sheets exhibited substantially higher first peak loads. Among the uniform helicoidal sandwich panels, S-H73(5) showed the highest first peak load of 8.87 kN and the highest second peak load of 7.62 kN, followed by S-H73(10) and S-H73(20). This indicates that a smaller inter-ply angle in the helicoidal face sheet is beneficial for enhancing both the local perforation resistance of the upper face sheet and the bending resistance of the lower face sheet. However, the improvement decreased as the inter-ply angle increased. S-H73(20) exhibited a first peak load of 7.89 kN and a second peak load of 4.96 kN, approaching the level of the conventional reference panels in the second loading stage. This suggests that excessively large inter-ply angles reduce the ability of the helicoidal architecture to redistribute damage through the thickness, especially during lower-face failure.

For the non-uniform helicoidal sandwich panels, the through-thickness distribution of the inter-ply angle had a pronounced effect on the second peak load and penetration energy. S-H73(5–10) reached the highest first peak load of 8.99 kN, but its second peak load decreased to 5.47 kN and its penetration energy was only 84.09 J. In contrast, S-H73(10–5) achieved a similarly high first peak load of 8.80 kN, a higher second peak load of 6.26 kN, and the highest penetration energy of 108.37 J among all tested sandwich panels. A similar trend was observed between S-H73(20–10) and S-H73(10–20) placing the smaller inter-ply angle near the upper loading side and the larger inter-ply angle near the lower tensile side led to improved second-stage load-bearing capacity and higher energy absorption.

The mass-normalized results in Table 8 further support the favorable overall performance of S-H73(10–5). This configuration achieved the highest SEA of 0.734 kJ/kg, representing increases of approximately 13.7% and 32.9% relative to S-CP73 and S-QI73, respectively. S-H73(5) and S-H73(20–10) also showed relatively high SEA values of 0.703 and 0.699 kJ/kg. Because all sandwich panels employed the same honeycomb geometry, dimensions, and constituent materials, the SEA values mainly reflect the influence of face-sheet layup on the specimen-level penetration response. However, these values are affected by the localized nature of penetration damage, since only a limited region beneath the indenter directly contributes to energy absorption. The energy-absorption-efficiency values provide additional information regarding load utilization during penetration, further indicating that peak-load utilization and total mass-specific energy absorption represent complementary performance characteristics.

These results suggest that the first peak load is mainly controlled by the local indentation and punching-shear resistance of the upper face sheet supported by the honeycomb core, whereas the second peak load is more strongly affected by the tensile-side damage resistance of the lower face sheet. Therefore, the optimal face-sheet layup for a sandwich panel is not necessarily the one that only maximizes the first peak load. Instead, a favorable configuration should maintain a high first peak load, sustain a stable core-crushing plateau, and delay lower-face failure. From this perspective, S-H73(10–5) provided the best overall balance between load-bearing capacity and energy absorption.

#### 3.3.2. Core Face-Sheet Interaction and Damage Morphology

**Figure 18:**
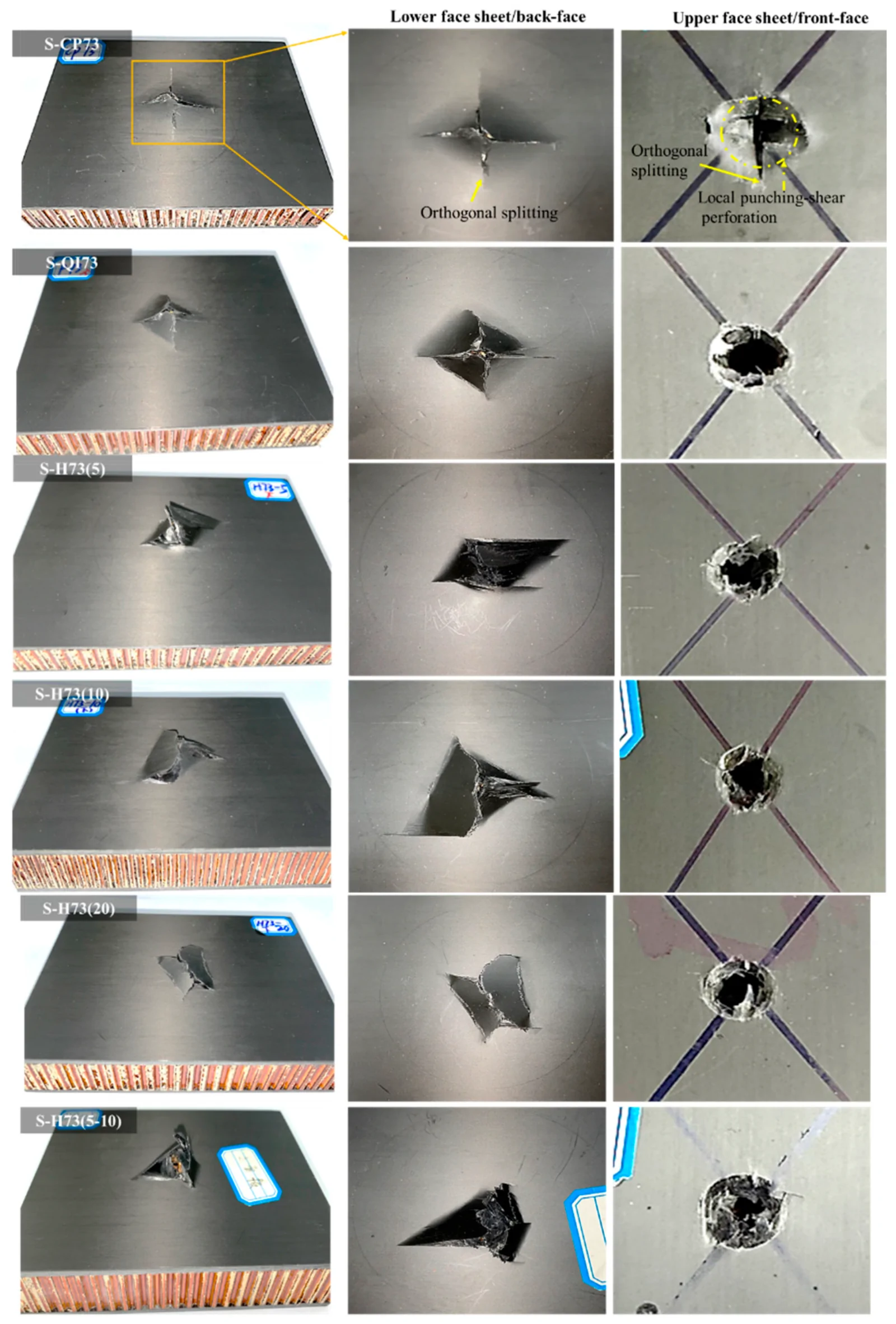

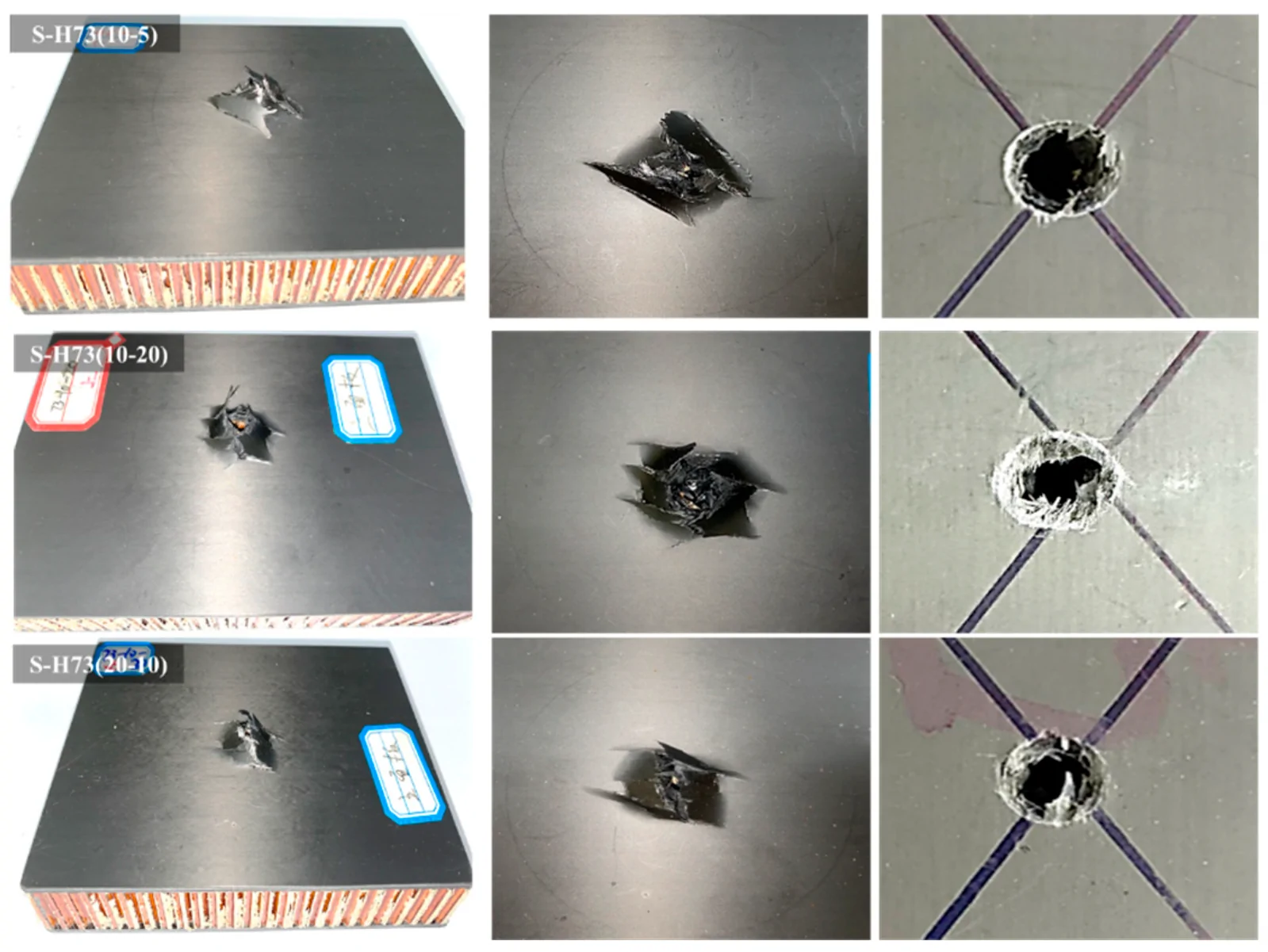
Post-test damage morphologies of the honeycomb sandwich panels after quasi-static penetration.

Figure 18 shows the post-test damage morphologies of representative sandwich panels. The upper loaded face sheets of all sandwich panels exhibited localized perforation around the indenter. The approximately circular holes indicate that the upper face sheet failed primarily through a punching-shear-dominated perforation mechanism assisted by local indentation. This local failure mode was caused by the high contact pressure and shear stress concentration around the hemispherical indenter and was accompanied by matrix crushing, fiber fracture, and local delamination.

Although the upper face sheets of all sandwich panels showed a similar local perforation zone, the secondary cracking patterns remained strongly layup-dependent. For S-CP73, cracks propagated preferentially along the 0° and 90° fiber directions, resulting in partially orthogonal splitting outside the perforation zone. In contrast, the helicoidal face sheets showed less distinct orthogonal cracking, suggesting that the through-thickness rotation of fiber orientation redistributed the crack path and reduced the tendency for damage to propagate along only two dominant fiber directions.

The lower face sheet exhibited damage morphologies similar to the back-face damage observed in the corresponding monolithic laminates. This is because the lower face sheet was not directly perforated at the beginning of loading. Instead, it failed after load transfer through the crushed honeycomb core, and its damage was therefore governed mainly by bending-induced tensile stresses, matrix splitting, fiber fracture, and delamination. Nevertheless, the sandwich configuration influenced the timing and severity of lower-face damage through core crushing and load redistribution. The lower-face damage should therefore be interpreted as a layup-controlled tensile-side failure coupled with core-mediated load transfer.

**Figure 19:**
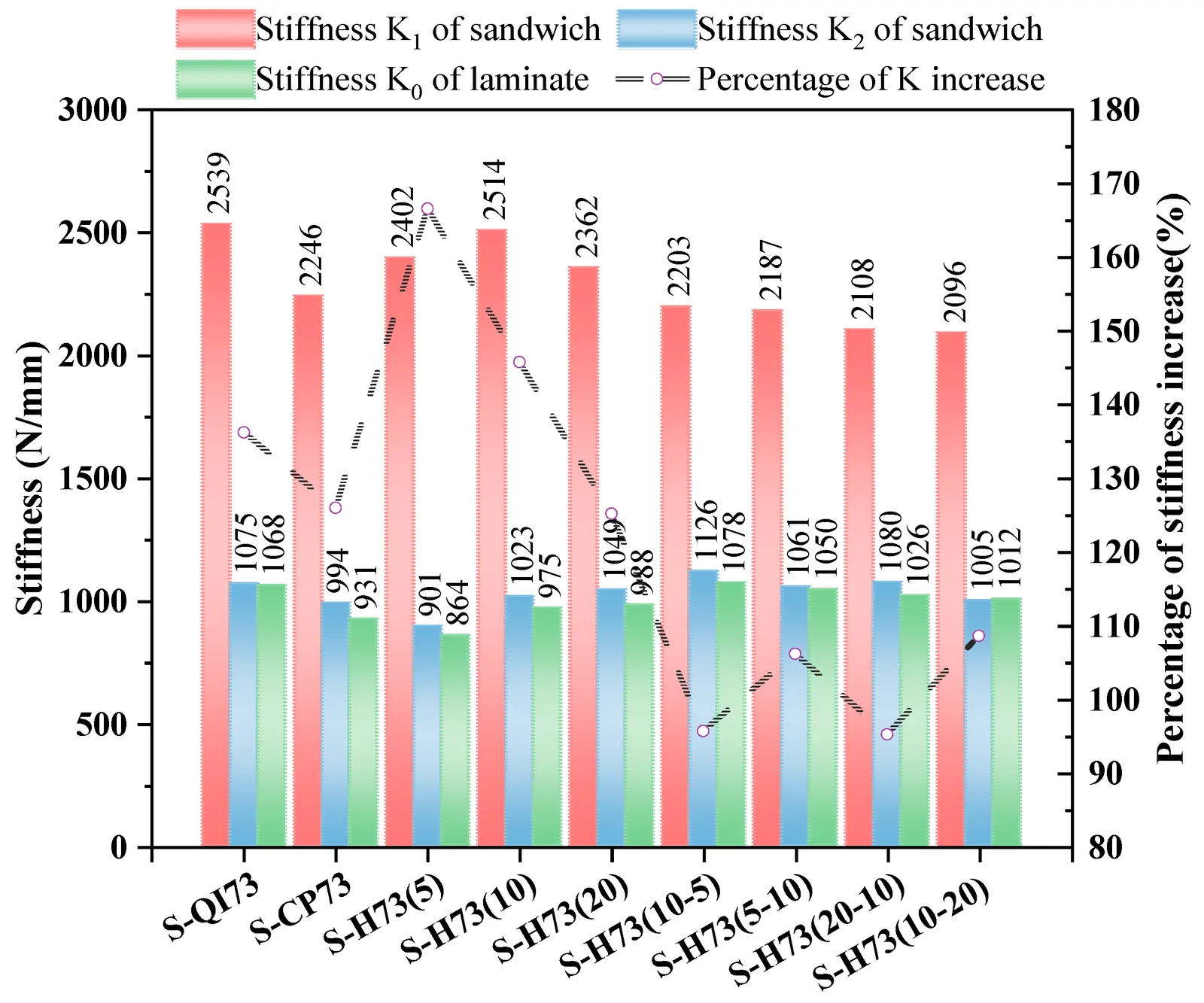
Comparison of the apparent stiffnesses of the monolithic laminates and honeycomb sandwich panels. The percentages indicate the increase in $K_{1}$ relative to $K_{0}$.

The stiffness comparison further confirms the two-stage load-bearing mechanism of the sandwich panels. As shown in Figure 19, the initial stiffness $K_{1}$ of the sandwich panels was much higher than the corresponding stiffness $K_{0}$ of the monolithic laminates, owing to the increased bending rigidity provided by the honeycomb core and the separation between the two face sheets. In contrast, the second-stage stiffness $K_{2}$, measured after upper-face perforation and during reloading toward lower-face failure, was closer to $K_{0}$. This indicates that once the upper face sheet was perforated and the core beneath the indenter was crushed, the lower face sheet became the primary load-bearing component. Table 9 reports the stiffness of each layup and the fitting statistics. The apparent first-stage stiffness $K_{1}$ exceeded the corresponding laminate stiffness $K_{0}$ by 104–178%, whereas $K_{2}$ differed from $K_{0}$ by less than approximately 7% for all configurations. This supports the transition from an intact sandwich response during the first loading stage to a lower-face-sheet-dominated response during the second reloading stage.

**Figure 20:**
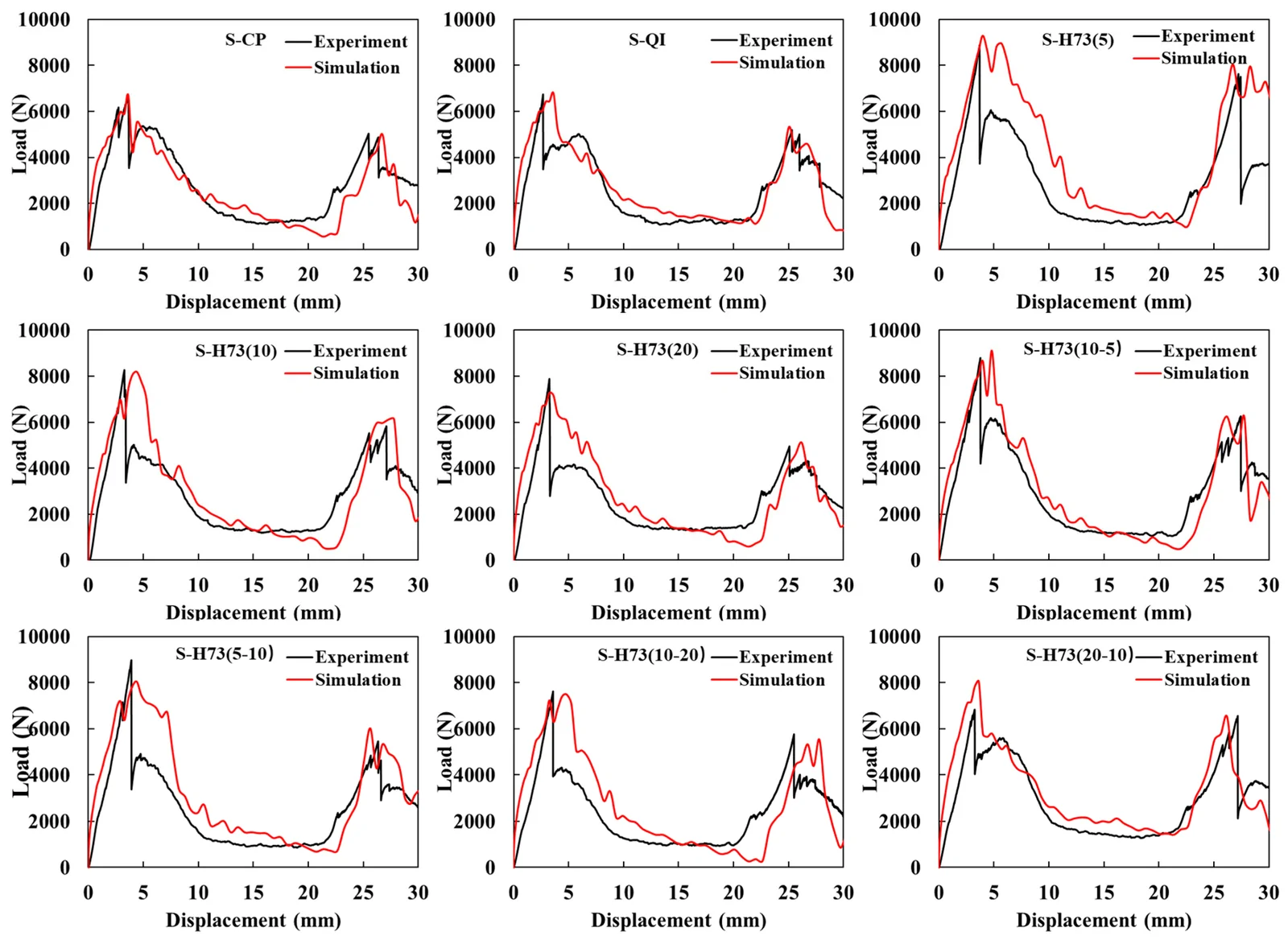
Comparison of the experimental and numerical load–displacement responses of the composite honeycomb sandwich panels under quasi-static penetration. The simulations reproduce the characteristic two-peak response, including upper-face-sheet perforation, the intermediate core-crushing plateau, and subsequent lower-face-sheet failure.

Finally, the finite element model was validated by comparing the simulated and experimental load–displacement responses of the sandwich panels, as shown in Figure 20. Overall, the numerical model reproduced the characteristic multi-stage response observed in the experiments, including the initial loading stage, the first peak load associated with upper-face-sheet perforation, the intermediate core-crushing plateau, the second peak load associated with lower-face-sheet failure, and the subsequent post-peak softening. Although deviations were observed in the pre-peak indentation stiffness, exact peak-load magnitudes, and post-peak fluctuations for some configurations, the simulations captured the essential two-peak response and the overall load-transfer sequence during quasi-static penetration. The pre-peak discrepancies may reflect the combined effects of the sublaminate discretization, the constitutive representation of the Nomex paper, the idealized face–core tie constraint, and the local contact treatment. In the experiments, the initial response of the sandwich panels was nearly linear, indicating that the early loading stage was governed mainly by elastic bending of the face sheets and elastic support from the honeycomb core. In the numerical model, however, local indentation beneath the hemispherical indenter, early core-wall deformation, and progressive damage initiation in the face sheets may occur before the first peak load, resulting in a more nonlinear pre-peak response. Therefore, the model is not interpreted as an exact point-by-point reproduction of the experimental curves but as a mechanism-oriented model capable of capturing the dominant deformation and failure sequence.

**Figure 21:**
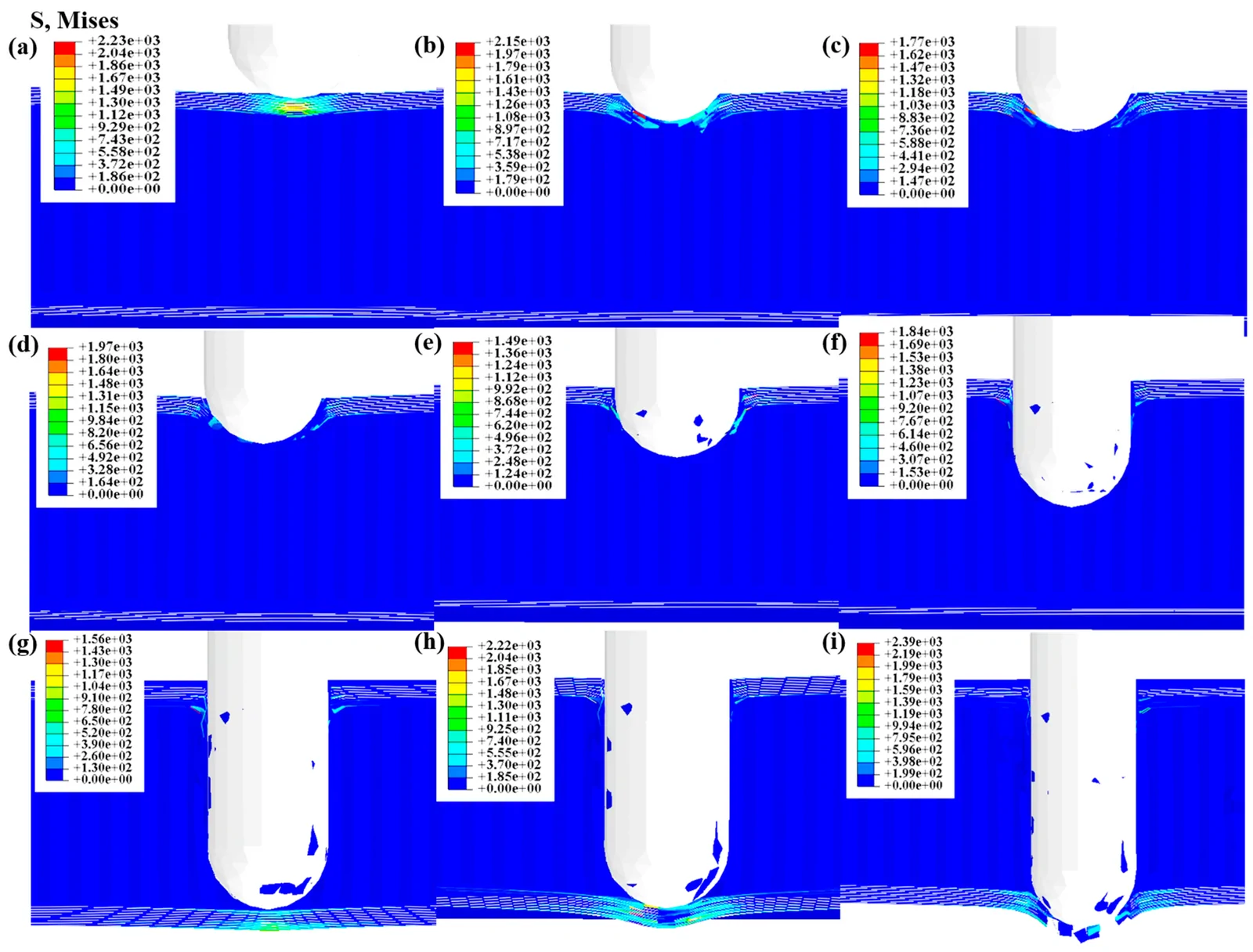
Simulated deformation and load-transfer sequence during quasi-static penetration of a honeycomb sandwich panel: (**a**–**i**) successive stages from upper-face-sheet indentation to final lower-face-sheet penetration. The unit of stress is MPa.

The simulated deformation sequence further clarifies the physical origin of the two-peak response, as illustrated in Figure 21. At the beginning of loading, the upper face sheet undergoes local indentation and bending while being supported by the honeycomb core. As the indentation depth increases, severe damage develops beneath the indenter and the upper face sheet reaches its local load-bearing limit, corresponding to the first peak load *P*_1_. After upper-face-sheet perforation, the load drops rapidly and then enters a relatively stable plateau. This plateau is governed primarily by progressive crushing and densification of the honeycomb core beneath the indenter. With further displacement, the crushed core transfers load to the lower face sheet. The lower face sheet then begins to deform under bending-dominated tensile loading, causing the load to rise again toward the second peak load P_2_. The second peak corresponds to the onset of major lower-face-sheet failure, followed by final penetration and residual post-peak load carrying. This sequential deformation process confirms that the sandwich-panel response is governed by two face-sheet failure events separated by a core-crushing stage.

**Figure 22:**
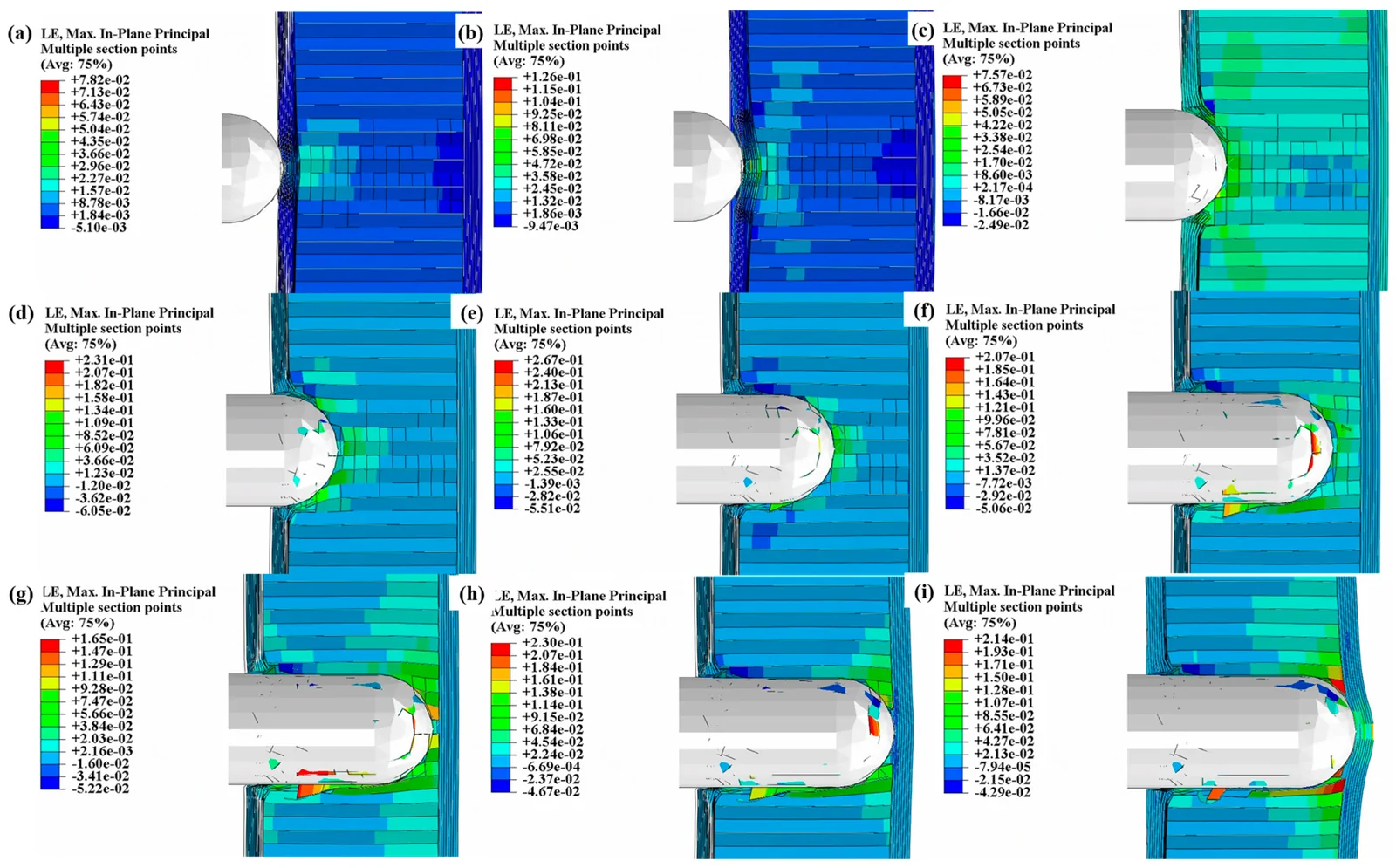
Evolution of local honeycomb cell-wall deformation during quasi-static penetration of a representative sandwich panel: (**a**–**i**) successive loading stages showing the maximum in-plane principal logarithmic strain in the explicitly modeled S4R honeycomb cell walls, from initial local deformation and progressive wall folding to core crushing, densification, and subsequent load transfer toward the lower face sheet.

To further resolve the local core response, Figure 22 shows the evolution of the explicitly modeled honeycomb cell walls at successive penetration stages. Local deformation first develops beneath the upper face sheet and becomes concentrated around the penetration zone after upper-face failure. With increasing displacement, the affected cell walls undergo progressive bending and buckling, followed by inward folding and contact between neighboring collapsed walls. The surrounding intact cell walls constrain the lateral expansion of the crushed region, thereby promoting localized collapse and the progressive accumulation of folded cell-wall material beneath the indenter. Continued compaction of this confined region leads to core densification and produces the characteristic core-crushing plateau observed in the load–displacement response. As the densified region becomes progressively stiffer, it provides an increasingly effective load-transfer path toward the lower face sheet, contributing to the subsequent rise toward the second load peak. Similar cell-wall buckling–folding–densification sequences have been reported for Nomex honeycomb cores under transverse compression and indentation [51]. In the present study, the honeycomb geometry and the relative position between the indenter and the underlying cell structure were held fixed for all sandwich configurations to isolate the effect of face-sheet layup. A systematic investigation of cell-center, cell-wall, and cell-junction loading positions was therefore not treated as an independent variable.

Overall, the model showed sufficient agreement with the experiments and was therefore used to interpret the internal damage mechanisms. In particular, the numerical results provide access to internal processes that cannot be fully resolved from experimental load–displacement curves and post-test photographs alone, including the interaction among upper-face-sheet perforation, honeycomb core crushing, interlaminar delamination, and lower-face-sheet failure.

### 3.4. Damage Mechanisms and Energy Absorption in Helicoidal Sandwich Panels

**Figure 23:**
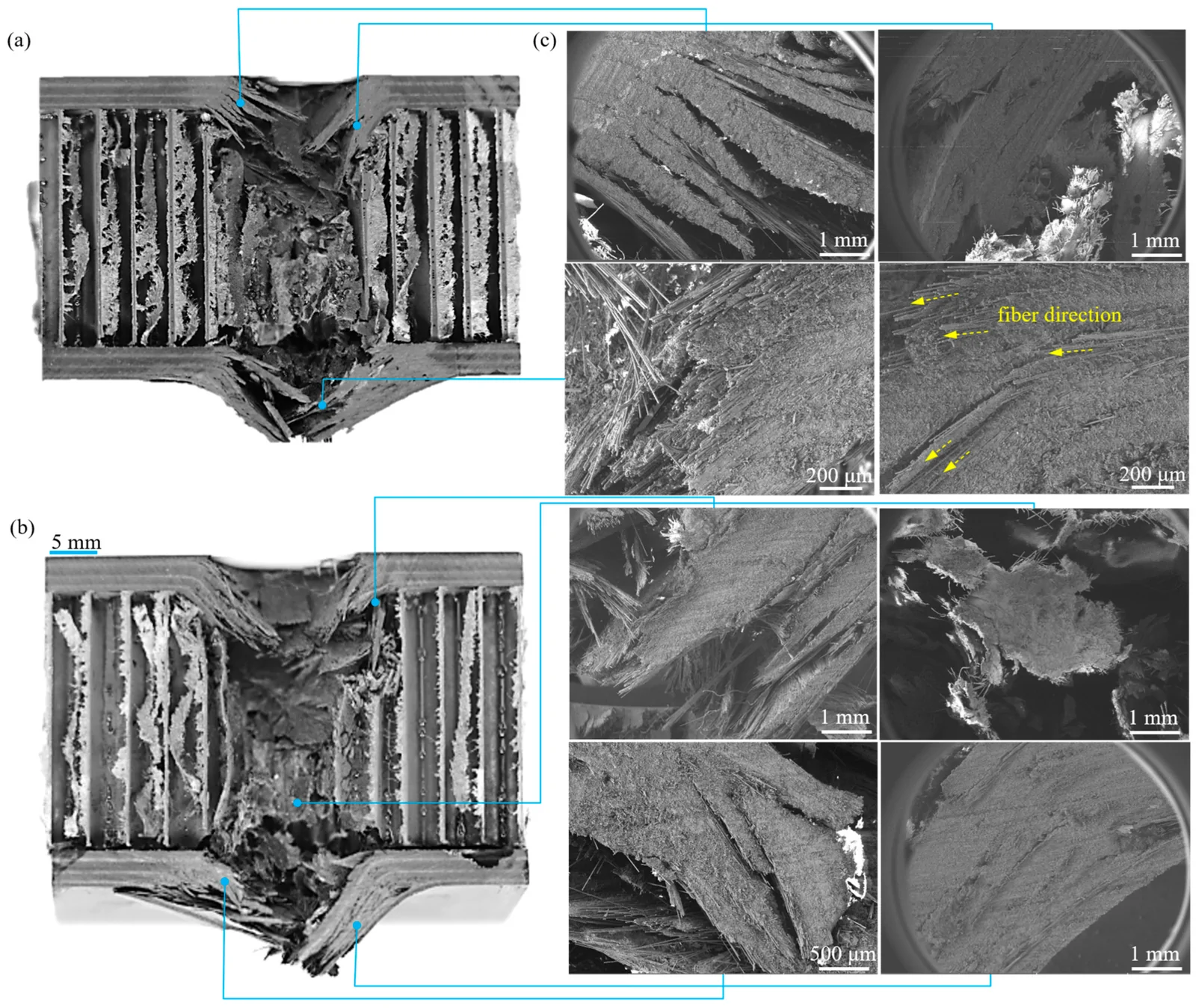
Cross-sectional and SEM observations of quasi-static penetration damage in representative helicoidal honeycomb sandwich panel: (**a**,**b**) cross-sectional views of specimen S-H73(10–5) showing localized upper-face-sheet perforation, honeycomb-core crushing and densification, and bending-dominated lower-face-sheet tearing; and (**c**) SEM micrographs of selected fracture regions showing fiber fracture, fiber pull-out, matrix cracking, interlaminar separation, and crushed core-wall fragments.

The damage mechanisms of the helicoidal sandwich panels differ from those of the monolithic laminates because the honeycomb core changes both the deformation mode of the face sheets and the load-transfer path during penetration. To further clarify the internal failure process, representative S-H73(10–5) specimens were sectioned and examined by scanning electron microscopy (SEM, TESCAN GAIA3), as shown in Figure 23. The cross-sectional observations reveal a highly localized damage zone beneath the indenter, where the upper face sheet was perforated, the honeycomb cell walls were severely crushed and fragmented, and the crushed core material accumulated into a compact densified region. This damage pattern corresponds well to the core-crushing plateau observed in the load–displacement response.

The SEM observations identify microscale fracture features associated with energy dissipation. Around the penetrated region, fiber fracture, fiber pull-out, matrix cracking, interlaminar separation, and crushed honeycomb-wall fragments can be observed. These features indicate that the penetration energy was dissipated by the coupled action of face-sheet fracture, interlaminar damage, and progressive honeycomb core crushing. In particular, the compact crushed core beneath the indenter suggests that the core does not simply lose its load-carrying function after upper-face perforation; instead, it forms a densified load-transfer region that continues to transmit force to the lower face sheet.

**Figure 24:**
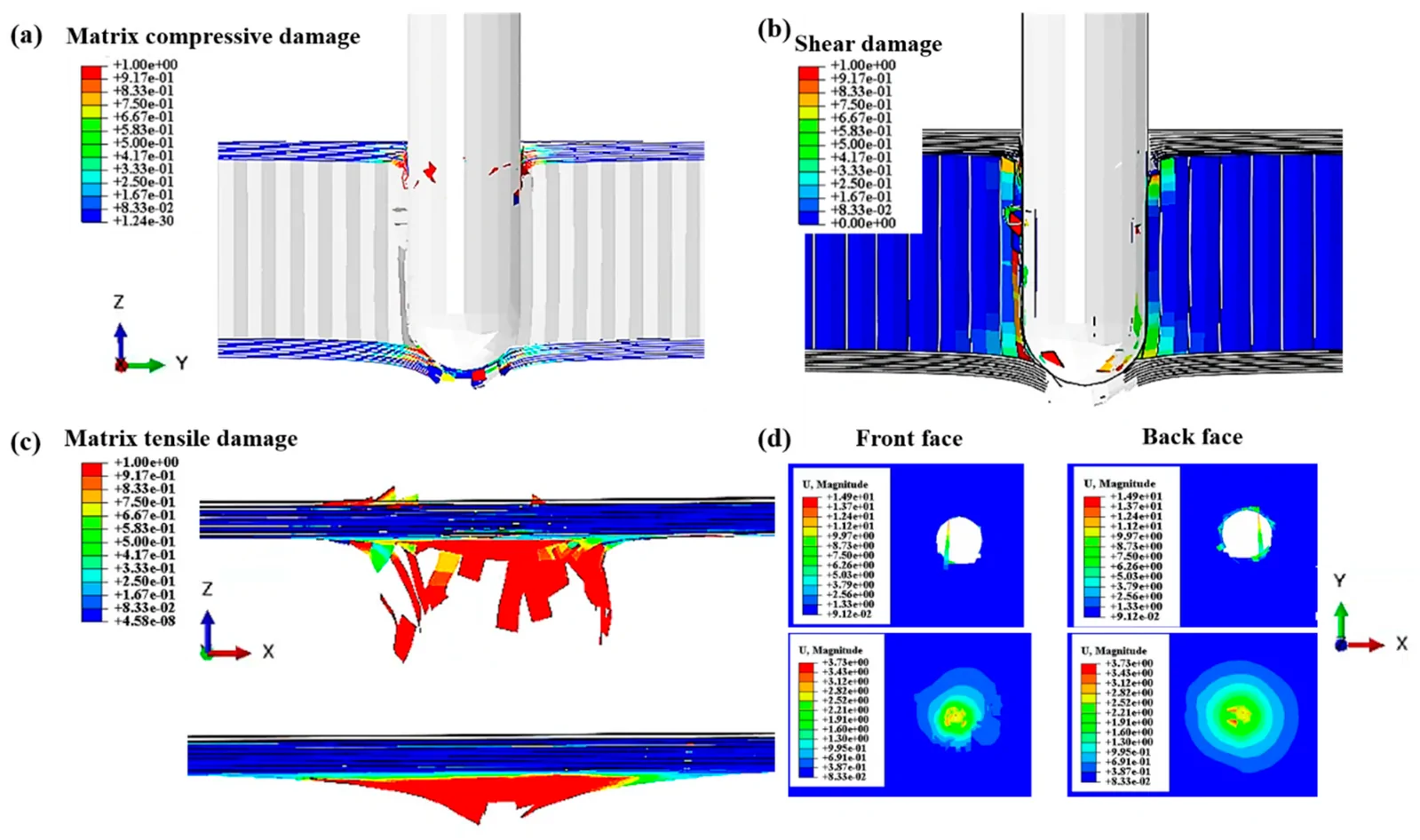
Numerically predicted damage and deformation of a representative honeycomb sandwich panel (S-QI73) under quasi-static penetration: (**a**) compressive damage in the composite face sheets; (**b**) shear-damage initiation criterion (SHRCRT) in the explicitly modeled aramid-paper honeycomb cell walls, where SHRCRT = 1 indicates satisfaction of the shear-damage initiation criterion; (**c**) tensile damage in the composite face sheets, showing localized upper-face perforation and bending- and membrane-dominated lower-face tearing; and (**d**) displacement-magnitude contours of the front and back face sheets.

The failure patterns of the upper and lower face sheets are also evident from the experimental observations in Figure 10 and Figure 18 and are further supported by the numerical results in Figure 24. The upper face sheet failed through a localized perforation mode, producing an approximately circular hole around the indenter. In contrast, the lower face sheet showed a more extensive tearing pattern governed by bending and tensile deformation.

For the monolithic laminates, the helicoidal layup improves penetration resistance through two coupled mechanisms. First, the gradual rotation of fiber orientations deflects matrix cracks and delamination paths, increasing the tortuosity of damage propagation. Second, the rotating ply orientations modify the anisotropic bending response and may introduce bending–twisting coupling. For the small-angle uniform helicoidal laminates, this response, together with progressive damage redirection, was associated with larger peak displacements than those of the conventional reference laminates. However, this deformation mechanism is substantially modified once the helicoidal laminate is used as the face sheet of a honeycomb sandwich panel. During the first loading stage, the intact honeycomb core acts as a continuous elastic foundation beneath the upper face sheet. This support increases the bending rigidity of the sandwich structure and strongly constrains the free bending and twisting deformation of the indented face sheet. As a result, the upper face sheet cannot fully develop the bending and twisting response observed in the monolithic helicoidal laminates. Instead, the deformation becomes concentrated beneath the hemispherical indenter, where local indentation, out-of-plane shear, matrix crushing, fiber fracture, and interlaminar damage develop. When these local damage modes coalesce around the indenter perimeter, the upper face sheet is perforated through an indentation-assisted punching-shear-dominated mechanism, giving rise to the first peak load $P_{1}$.

The constrain of core makes the first-stage stiffness $K_{1}$ of the sandwich panels much higher than the stiffness $K_{0}$ of the corresponding monolithic laminates, while the differences among different face-sheet layups become less pronounced. The monolithic-laminate stiffness reflects the layup-dependent bending and indentation response. Core support reduces the divergence among $K_{1}$ of the helicoidal configurations and shifts the early response toward a combined face-sheet/core structural behavior.

After the perforation of the upper face sheet, the load drops sharply and the penetration process enters a core-dominated stage. The honeycomb cell walls beneath the indenter undergo severe folding, shear deformation, and progressive crushing. The crushed core does not simply lose its load-bearing function; instead, it becomes locally densified and forms a compact crushed zone beneath the indenter. This densified core region sustains the plateau load and transfers the concentrated penetration force toward the lower face sheet. Once the densified core engages the lower face sheet, the lower face sheet becomes the primary load-bearing component, and the load rises again toward the second peak $P_{2}$.

**Figure 25:**
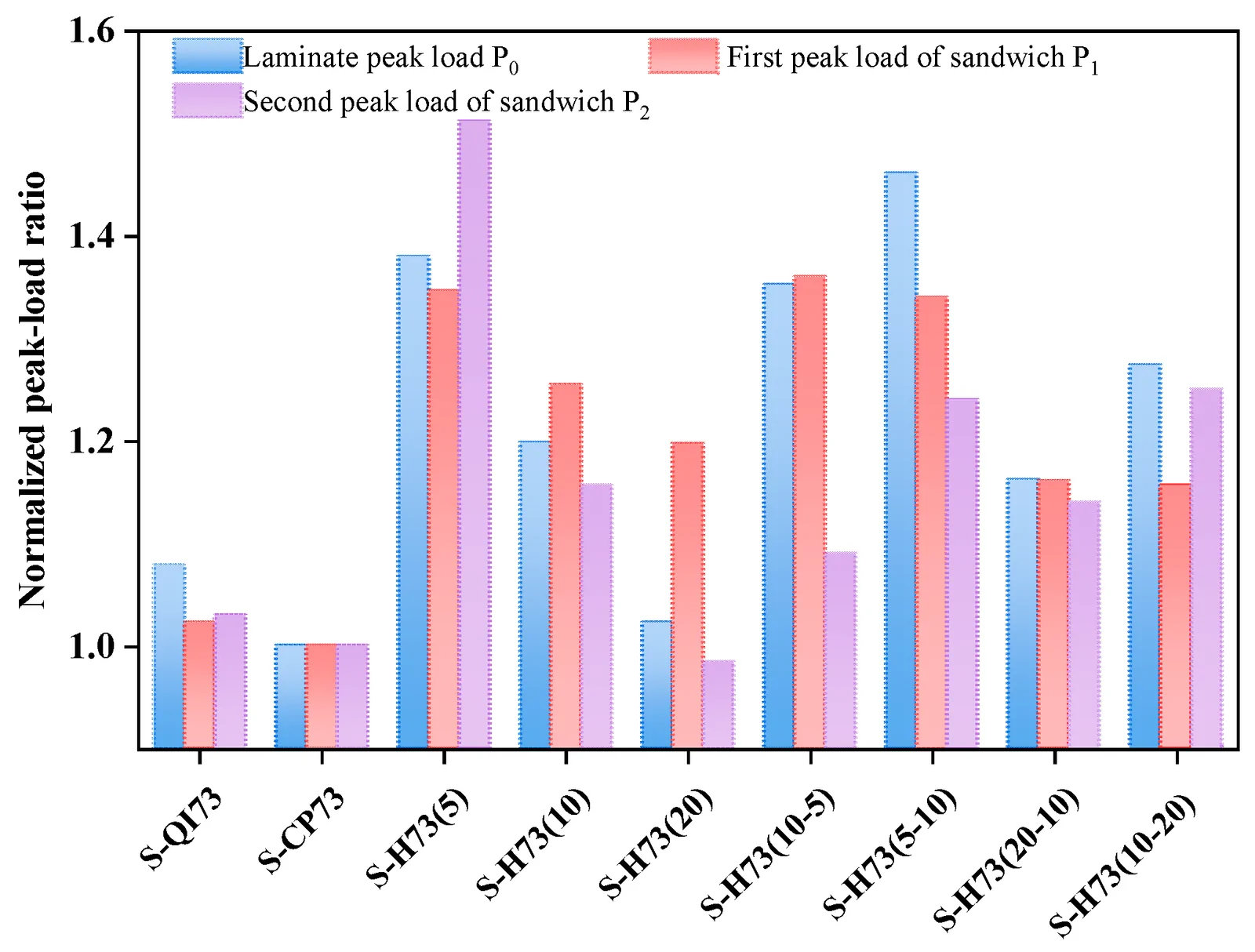
Normalized peak-load ratios of the monolithic laminates and honeycomb sandwich panels. The plotted quantities are $P_{0}/P_{0,CP73}$, $P_{1}/P_{1,S-CP73}$, and $P_{2}/P_{2,S-CP73}$, where $P_{0}$ is the peak load of a monolithic laminate and $P_{1}$ and $P_{2}$ are the first and second peak loads of the corresponding sandwich panel, respectively.

The lower face sheet fails under a loading condition that is much closer to that of the monolithic laminate. Unlike the upper face sheet, it is not backed by an intact elastic core beneath the indenter; instead, it spans the unsupported circular opening and deforms through global bending and membrane stretching. Therefore, its failure morphology retains the layup-dependent characteristics identified in the monolithic laminate tests and in the numerical interpretation in Section 3.2. Cross-ply face sheets tend to produce orthogonal splitting along the principal fiber directions, whereas helicoidal face sheets promote crack deflection and more tortuous tearing paths. The lower-face failure should therefore be interpreted as bending-dominated tensile tearing mediated by the crushed core, rather than as a simple punching-shear failure. The normalized peak-load comparison in Figure 25 provides further evidence for this interpretation. The normalized second peak load $P_{2}$ follows a trend broadly consistent with the peak load $P_{0}$ of the corresponding monolithic laminates. For instance, QI73 is higher than CP73, the uniform helicoidal laminates follow the order H73(5) > H73(10) > H73(20), and the hybrid configurations show the same sequence effect, with H73(10–5) exceeding H73(5–10) and H73(20–10) exceeding H73(10–20). This consistency indicates that $P_{2}$ is strongly influenced by the lower-face-sheet layup, although the densified core and the evolving contact conditions also contribute to the measured peak. By contrast, the normalized first peak load $P_{1}$ exhibits a more compressed distribution among different helicoidal sandwich panels. Although the uniform helicoidal panels still follow the order S-H73(5) > S-H73(10) > S-H73(20), the differences are smaller than those observed for the monolithic laminates and for $P_{2}$. For the hybrid configurations, the sequence effect becomes even less pronounced, and S-H73(5–10) shows a first peak load comparable to, or slightly higher than, S-H73(10–5). This indicates that $P_{1}$ is not a direct reflection of the monolithic laminate peak load. Instead, it is governed by the local upper-face perforation process under core support. The honeycomb core restricts the free bending and twisting of the upper face sheet, thereby reducing the sensitivity of $P_{1}$ to the detailed through-thickness layup sequence.

**Figure 26:**
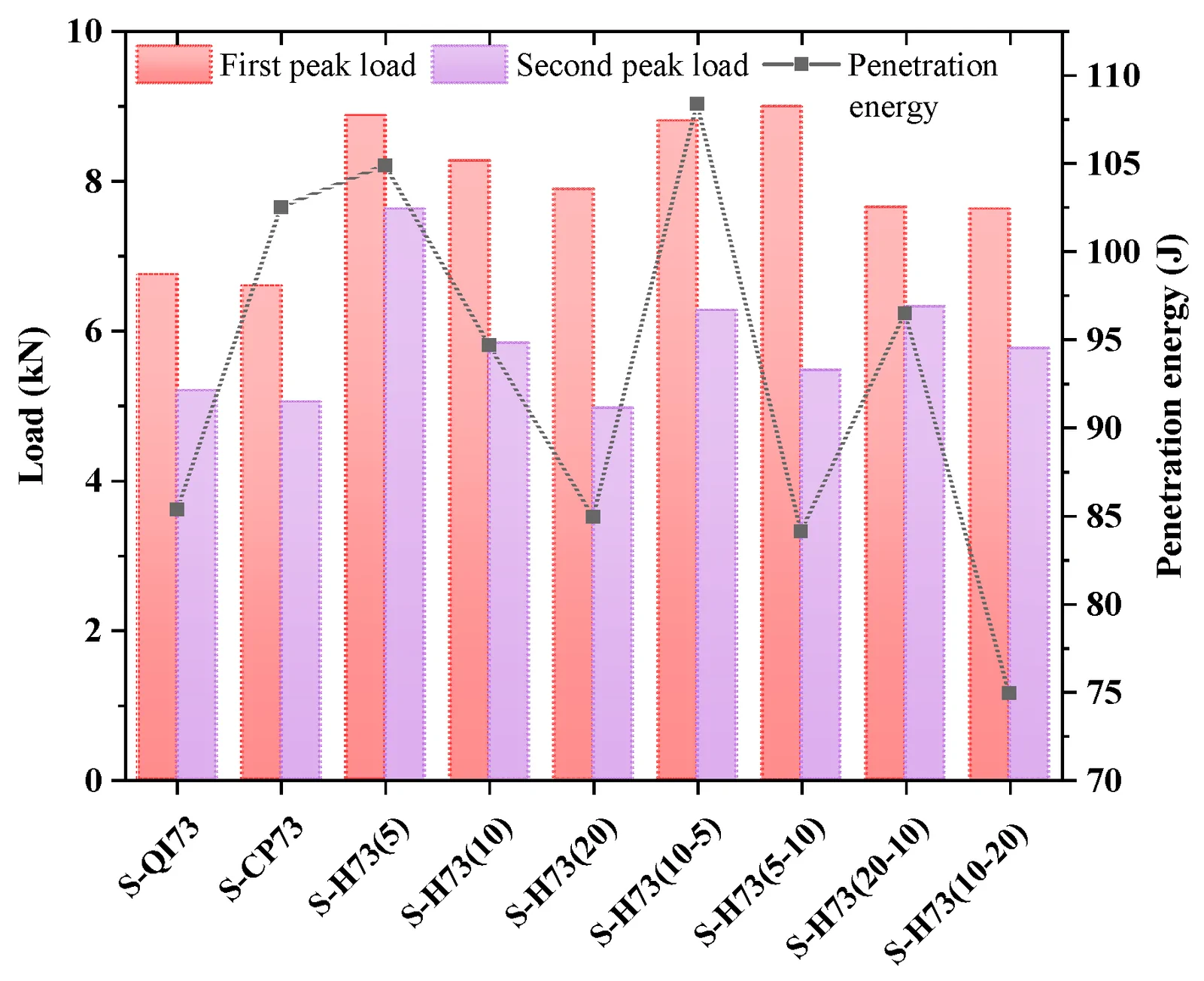
Peak loads and penetration energies of the tested honeycomb sandwich panels.

The peak-load and energy results in Figure 26 show that helicoidal face sheets produce pronounced layup-dependent changes in the peak loads and penetration energy of the sandwich panels. Among the uniform helicoidal configurations, S-H73(5) achieved the highest first and second peak loads, indicating that a smaller inter-ply angle is more effective in maintaining a continuous crack-deflection path and resisting both upper-face perforation and lower-face tearing. As the inter-ply angle increased from 5° to 20°, both peak loads and penetration energy decreased, suggesting that excessively large pitch angles weaken the gradual damage-deflection mechanism of the helicoidal architecture. For the hybrid configurations, however, the highest penetration energy was obtained by S-H73(10–5), even though it did not exhibit the highest individual $P_{1}$ or $P_{2}$. This result indicates that energy absorption is governed by the entire penetration history rather than by a single peak load. The S-H73(10–5) sandwich panel therefore achieved a favorable balance among local upper-face perforation resistance, lower-face bending resistance, and sustained load carrying during core crushing. The high penetration energy of this configuration resulted from this balanced load-bearing response over the entire penetration process, rather than from the maximization of either $P_{1}$ or $P_{2}$ alone.

**Figure 27:**
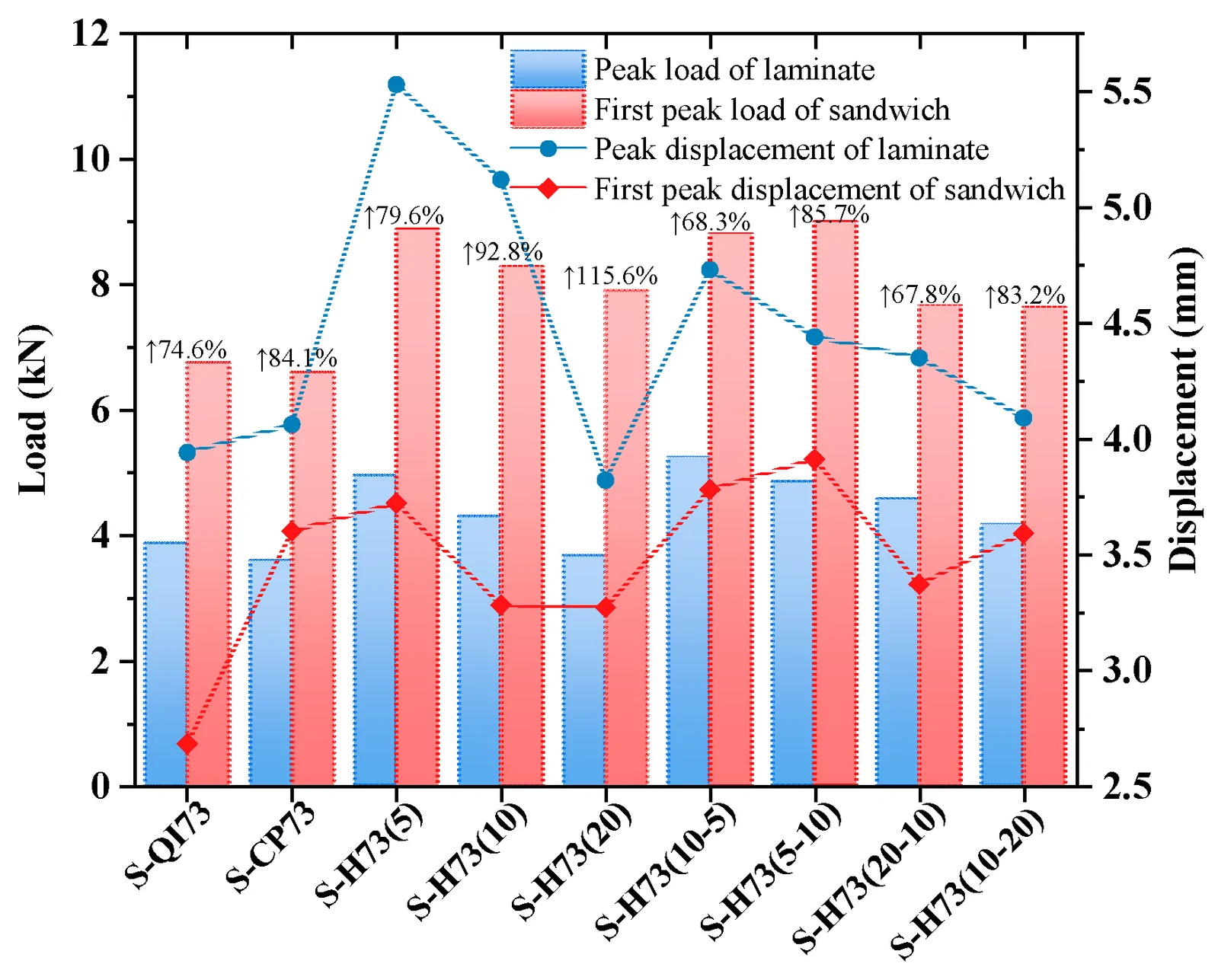
Comparison of the peak loads and corresponding displacements of the monolithic laminates and honeycomb sandwich panels. $P_{0}$ denotes the peak load of a monolithic laminate, while $P_{1}$ denotes the first peak load of the corresponding sandwich panel. The percentages indicate the increase in $P_{1}$ relative to $P_{0}$.

The role of honeycomb support is further illustrated in Figure 27. Compared with the corresponding monolithic laminates, all sandwich panels show substantially higher first peak loads, confirming the stiffening effect of the honeycomb core and the increased bending rigidity generated by separating the two face sheets. At the same time, the displacement at the first peak is reduced. This indicates that the core support increases the local load-bearing capacity of the upper face sheet but also promotes earlier localized perforation by suppressing the large bending and bending–twisting deformation that would otherwise occur in the monolithic laminate. For the uniform helicoidal configurations, the absolute first peak load decreases from S-H73(5) to S-H73(20), whereas the relative peak-load amplification caused by the honeycomb core increases (from 79.6% to 115.6%). This is because the monolithic H73(20) laminate has a relatively low peak load; thus, the structural contribution of the honeycomb core becomes more prominent in relative terms.

Overall, the enhanced penetration resistance of the helicoidal honeycomb sandwich panels results from a coupling between laminate-level toughening and sandwich-level load transfer. At the laminate level, the helicoidal architecture redirects damage through multiple orientations and modifies the anisotropic bending response. At the sandwich level, the honeycomb core constrains the global deformation of the upper face sheet, increases the first-stage stiffness, induces localized punching-shear perforation of the upper face sheet, absorbs energy through progressive core crushing, and transfers load to the lower face sheet. The final performance of the sandwich panel is therefore governed not simply by the intrinsic strength of the face-sheet laminate but by the compatibility between face-sheet layup, core support, and the sequential upper-core-lower load-transfer process. This explains why the H73(10–5) configuration, although not maximizing every individual peak-load metric, exhibits the highest overall penetration energy among the tested sandwich panels.

## 4. Conclusions

This study shows that the penetration resistance of helicoidal laminates is governed not by the inter-ply angle alone but by how the repeated angular increment organizes damage through the thickness. A change from 5° to 10° doubles the rate of fiber-orientation rotation between successive plies and therefore alters the directional continuity of damage over many interfaces. Smaller angular increments reduce inter-ply mismatch and suppress delamination but allow matrix splits to propagate over longer distances along gradually changing material directions. Moderate rotation of fiber redirects the preferred damage direction more rapidly and interrupts this splitting while retaining progressive crack deflection; excessively large rotation, in contrast, increases inter-ply mismatch and promotes delamination and more localized ply-block or flap-like failure. This competition explains the non-monotonic dependence on pitch angle: H73(5) carried the highest peak load among the uniform helicoidal laminates, whereas H73(10) dissipated the most penetration energy because damage could develop over multiple orientations before a continuous failure path formed.

The hybrid results further show that through-thickness placement of the pitch angle is as important as its magnitude. Delamination develops preferentially toward the loaded side, whereas matrix splitting is driven primarily from the tensile side. H73(10–5) therefore places the smaller 5° increment where resistance to delamination is most beneficial and the larger 10° increment where interruption of long matrix splits is required. This spatial matching of layup architecture to the local damage mode, rather than simple averaging of two pitch angles, explains why H73(10–5) reached the highest laminate peak load of 5.23 kN, 46.1% above CP73 and 35.5% above QI73. The result suggests a more general design principle for graded helicoidal laminates: the local rotation rate should be selected according to the dominant damage mechanism expected in each through-thickness region.

Introducing the honeycomb core changes this competitive damage balance by reorganizing both deformation and load transfer. The intact core suppresses unconstrained bending of the upper face sheet and promotes localized indentation-assisted punching-shear perforation, producing the first load peak. After upper-face penetration, progressive core crushing and densification provide an intermediate energy-dissipation and load-transfer stage before the lower face sheet becomes dominant and fails through bending- and membrane-controlled tensile tearing. The different loading environments of the two face sheets explain why different layups maximize the first peak, second peak, and total penetration energy. S-H73(5–10) produced the highest first peak load, whereas S-H73(10–5) maintained a comparably high first-stage resistance together with stronger subsequent load bearing and achieved the highest penetration energy of 108.37 J. Thus, the optimum monolithic-laminate performance cannot be transferred directly to the sandwich structure. Face-sheet design should instead account for the complete sequential load path (upper-face perforation, core crushing and densification, and lower-face failure) rather than maximizing a single peak-load metric.

The sandwich simulations reproduced the characteristic two-peak response and the principal damage sequence, although deviations remained in the pre-peak response and peak magnitudes for some configurations. The eight-sublaminate representation does not resolve every ply-level delamination event, and the idealized face–core tie constraint excludes face–core debonding. The conclusions are therefore specific to the tested face-sheet thickness, honeycomb density, indenter and support geometry, and quasi-static loading condition. Future work should quantify face–core debonding, examine additional core densities and face-sheet thicknesses, assess whether the identified hybrid-angle sequence remains effective under low-velocity impact, and employ digital image correlation to characterize full-field deformation and strain localization during penetration.

## Figures and Tables

**Table 1:** Bouligand-structured laminate and sandwich-panel configurations and specimen designations.

Type	Designation	Description	Configuration/Ply Orientation (°)
Laminate	H73(5)	73-ply double helicoidal, 5° inter-ply angle	[0/5/10…/360]
	H73(10)	73-ply quadruple helicoidal, 10° inter-ply angle	[0/10/20…/720]
	H73(20)	73-ply octuple helicoidal, 20° inter-ply angle	[0/20/40…/1440]
	H73(5–10)	73-ply helicoidal, 10° inter-ply angle for upper half plies; 5° inter-ply angle for bottom half plies	[0/5/10…/175/180/190/200…/530/540]
	H73(10–5)	73-ply helicoidal with 5° inter-ply angle for upper half plies; 10° inter-ply angle for bottom half plies	[0/10/20…/350/360/365/370…/535/540]
	H73(10–20)	73-ply helicoidal with 20° inter-ply angle for upper half plies; 10° inter-ply angle for bottom half plies	[0/10/20…/350/360/380/400…/1060/1080]
	H73(20–10)	73-ply helicoidal with 10° inter-ply angle for upper half plies; 20° inter-ply angle for bottom half plies	[0/20/40…/700/720/730/740…/1070/1080]
	CP73	73-ply cross-ply	[(0/90)_36_/0]
	QI73	73-ply quasi-isotropic	[(0/45/90/−45)_9_/0/(−45/90/45/0)_9_]
Sandwich	S-H73(5)	The upper and lower face sheets are 73-ply laminates bonded to a honeycomb core.	H73(5)–honeycomb core–H73(5)
	S-H73(10)		H73(10)–honeycomb core–H73(10)
	S-H73(20)		H73(20)–honeycomb core–H73(20)
	S-H73(5–10)		H73(5–10)–honeycomb core–H73(5–10)
	S-H73(10–5)		H73(10–5)–honeycomb core–H73(10–5)
	S-H73(10–20)		H73(10–20)–honeycomb core–H73(10–20)
	S-H73(20–10)		H73(20–10)–honeycomb core–H73(20–10)
	S-CP73		CP73–honeycomb core–CP73
	S-QI73		QI73–honeycomb core–QI73

**Table 2:** Thicknesses of the laminates, honeycomb core, and assembled sandwich panels.

Type	Designation	Thickness			Specimen Mass/g
		Nominal ply/μm	Core/mm	Total/mm	
Laminate	H73(5)	30	/	2.2	49.6
	H73(10)				55.5
	H73(20)				56.6
	H73(5–10)				57.2
	H73(10–5)				57.3
	H73(10–20)				57.1
	H73(20–10)				56.9
	CP73				58.5
	QI73				56.0
Sandwich	S-H73(5)	30	20	24.4	149.3
	S-H73(10)				154.8
	S-H73(20)				152.1
	S-H73(5–10)				149.3
	S-H73(10–5)				147.6
	S-H73(10–20)				139.1
	S-H73(20–10)				138.1
	S-CP73				158.8
	S-QI73				154.5

**Table 3:** Summary of the finite element model definitions.

Component	Element Type	Model Description	Damage Model
Face-sheet	SC8R	continuum shell layer	2D Hashin damage degradation
Interlaminar interface	COH3D8	traction–separation model	quadratic stress and BK mixed-mode energy
Honeycomb core	S4R	explicit cell-wall shell model	Elastoplastic response with shear-damage initiation and evolution
Indenter	R3D4	rigid hemispherical indenter	rigid body
Support fixture	R3D4	rigid cylindrical support	fully constrained

**Table 5:** Experimental quasi-static penetration parameters of the 73-ply laminate specimens.

Type	Designation	Peak Load/kN	Displacement at Peak Load/mm	Penetration Energy/J	Failure Displacement/mm	SEA/kJ·kg^−1^	$\eta_{E}$/%
laminate	QI73	3.86	3.94	7.14	3.94	0.128	46.9
	CP73	3.58	4.06	10.02	5.10	0.171	54.9
	H73(5)	4.94	5.53	9.75	5.53	0.197	35.7
	H73(10)	4.29	5.12	10.55	5.12	0.190	48.0
	H73(20)	3.66	3.82	9.23	4.76	0.163	53.0
	H73(5–10)	4.84	4.44	8.11	4.45	0.142	37.7
	H73(10–5)	5.23	4.73	9.34	4.73	0.163	37.8
	H73(10–20)	4.16	4.09	10.22	5.02	0.179	48.9
	H73(20–10)	4.56	4.35	9.68	4.66	0.170	45.6

**Table 6:** Apparent initial stiffness of the 73-ply laminate specimens obtained from load–displacement response.

Type	Designation	Stiffness K_0_ (N/mm)	Statistical Parameter *R*^2^
laminate	QI73	1068	0.990
	CP73	931	0.994
	H73(5)	864	0.938
	H73(10)	975	0.984
	H73(20)	988	0.990
	H73(5–10)	1050	0.970
	H73(10–5)	1078	0.967
	H73(10–20)	1012	0.989
	H73(20–10)	1026	0.988

**Table 7:** Experimental quasi-static penetration parameters of the composite honeycomb sandwich panels.

Type	Designation	Peak Load /kN		Peak Displacement /mm		Plateau Load /kN	Final Penetration Displacement /mm
		First	Second	First	Second		
Sandwich	S-QI73	6.74	5.19	2.68	25.32	1.20	34.18
	S-CP73	6.59	5.04	3.63	25.51	1.21	38.65
	S-H73(5)	8.87	7.62	3.72	27.25	1.14	37.07
	S-H73(10)	8.27	5.83	3.28	27.10	1.28	34.20
	S-H73(20)	7.89	4.96	3.27	25.08	1.37	33.45
	S-H73(10–5)	8.80	6.26	3.78	27.46	1.18	34.93
	S-H73(5–10)	8.99	5.47	3.91	26.34	0.95	34.13
	S-H73(20–10)	7.65	6.55	3.37	26.24	1.28	36.48
	S-H73(10–20)	7.62	5.76	3.59	25.49	1.02	32.15

**Table 8:** Penetration energy, specific energy absorption, and energy absorption efficiency of the composite honeycomb sandwich panels.

Designation	Mass/g	Penetration Energy/J	SEA/kJ·kg^−1^	$\eta_{E}$/%
S-QI73	154.5	85.33	0.552	37.0
S-CP73	158.8	102.52	0.646	40.3
S-H73(5)	149.3	104.90	0.703	31.9
S-H73(10)	154.8	94.67	0.612	33.5
S-H73(20)	152.1	84.91	0.558	32.2
S-H73(10–5)	147.6	108.37	0.734	35.3
S-H73(5–10)	149.3	84.09	0.563	27.4
S-H73(20–10)	138.1	96.48	0.699	34.6
S-H73(10–20)	139.1	74.92	0.539	30.6

**Table 9:** Apparent first and second stage stiffnesses of the honeycomb sandwich panels with different face-sheet layups.

Type	Designation	Stiffness K_1_ (N/mm)	Statistical Parameter *R*^2^	Stiffness K_2_ (N/mm)	Statistical Parameter *R*^2^
Sandwich	S-QI73	2539	0.994	1075	0.974
	S-CP73	2246	0.985	994	0.992
	S-H73(5)	2402	0.997	901	0.983
	S-H73(10)	2514	0.996	1023	0.989
	S-H73(20)	2362	0.996	1049	0.995
	S-H73(10–5)	2203	0.997	1126	0.990
	S-H73(5–10)	2187	0.996	1061	0.990
	S-H73(20–10)	2108	0.997	1080	0.975
	S-H73(10–20)	2096	0.994	1005	0.977

## Data Availability

The original contributions presented in this study are included in the article. Further inquiries can be directed to the corresponding author.
